# Time series and power law analysis of crop yield in some east African countries

**DOI:** 10.1371/journal.pone.0287011

**Published:** 2023-06-13

**Authors:** Idika E. Okorie, Emmanuel Afuecheta, Saralees Nadarajah

**Affiliations:** 1 Department of Mathematics, Khalifa University, Abu Dhabi, UAE; 2 Department of Mathematics and Statistics, King Fahd University of Petroleum & Minerals, Dhahran, Saudi Arabia; 3 Interdisciplinary Research Center for Finance and Digital Economy, KFUPM, Dhahran, Saudi Arabia; 4 Department of Mathematics, University of Manchester, Manchester, United Kingdom; University of Siena, ITALY

## Abstract

We carry out a time series analysis on the yearly crop yield data in six east African countries (Burundi, Kenya, Somalia, Tanzania, Uganda and Rwanda) using the autoregressive integrated moving average (ARIMA) model. We describe the upper tail of the yearly crop yield data in those countries using the power law, lognormal, Fréchet and stretched exponential distributions. The forecast of the fitted ARIMA models suggests that the majority of the crops in different countries will experience neither an increase nor a decrease in yield from 2019 to 2028. A few exceptional cases correspond to significant increase in the yield of sorghum and coffee in Burundi and Rwanda, respectively, and significant decrease in the yield of beans in Burundi, Kenya and Rwanda. Based on Vuong’s similarity test *p*–value, we find that the power law distribution captured the upper tails of yield distribution better than other distributions with just one exceptional case in Uganda, suggesting that these crops have the tendency for producing high yield. We find that only sugar cane in Somalia and sweet potato in Tanzania have the potential of producing extremely high yield. We describe the yield behaviour of these two crops as black swan, where the “*rich getting richer*” or the “*preferential attachment*” could be the underlying generating process. Other crops in Burundi, Kenya, Somalia, Tanzania, Uganda and Rwanda can only produce high but not extremely high yields. Various climate adaptation/smart strategies (use of short-duration pigeon pea varieties, use of cassava mosaic disease resistant cassava varieties, use of improved maize varieties, intensive manuring with a combination of green and poultry manure, early planting, etc) that could be adapted to increase yields in east Africa are suggested. The paper could be useful for future agricultural planning and rates calibration in crop risk insurance.

## 1 Introduction

Africa is the poorest continent. It is struggling to feed its people. Hence, enhancement of crop production is important.

Furthermore, farmers are more interested in investing in crops that are capable of producing high yields not crops that can produce extremely low yield. They want to maximize the profit on their investment. Crops that have the potential for high yield are likely to attract low premium in crop yield insurance.

There have been several papers on high crop yield in African countries. While discussing nutrients in the west African Sudano-Sahelian zone, [1] noted that “shrubs and trees with their alternating periods of nutrient storing and recycling in leaves and wood, micro-depressions, termite mounts and ant nests become localised points of nutrient concentration and high crop productivity”. While investigating the importance of liming acid soils, [2] demonstrated that “severely acidified soils of the western highlands of Cameroon should be limed at moderate rates to sustain crop productivity”. While examining the seed supply system for maize production in southwestern Nigeria, [3] observed that “about 39% of farmers used improved varieties for high crop yields, 24% for disease resistance and 22% for market preferences, whereas local varieties were cultivated by 37% of farmers because of market preferences and availability, 16% because of low cost and 12% because of disease resistance”. [4] demonstrated that continuous-flow drip irrigation in Bauchi state of Nigeria delivers “high crop yields especially if the crops are grown under appropriate agronomic practices that enable protraction of the growth season”. [5] demonstrated that high maize yields on sandy soils in Zimbabwe can be achieved by using mineral fertilizers. According to [6], among many oilseed crops (for example, sunflower, soybeans, rapeseed/mustard, sesame, groundnuts, etc) grown in Kenya, oilseed rape is preferred because of its high yields (1.5 tons—4.0 tons / hectare) with high oil content of 42–46%. While comparing three fertigation strategies of grapes in the Berg River Valley region of South Africa, [7] found that “less berry crack contributed to a higher yield and higher export percentage of grapes”. While analysing the benefits of soil conservation in the Kondoa eroded area of Tanzania by conducting a household survey of 240 households, [8] observed that 56% of the respondents gained high crop yields. [9] investigated limited nitrogen content, a major challenge to sustainable and high crop production, for agricultural soils of lower eastern Kenya. While evaluating small holder farmers’ preferences for climate smart agricultural practices in Tehuledere district, northeastern Ethiopia, [10] found that “high and moderate climate resilience and high crop yield agricultural practices had a positive utility”. [11] demonstrated that phosphorus treatment for rice fields in lowlands in the central highlands of Madagascar significantly and consistently accelerated initial production with high crop growth rate and shortened days to heading. According to [12], “rain fed agriculture has a high crop yield potential if rainfall and soil nutrient input resources are utilized effectively”.

But none of these papers discuss the distribution of crop yield or forecasts. The distributions of crop yields is very useful in agribusiness. These distributions can help to tackle food shortages and insecurity by understanding how natural resources and farmers attitude towards crops selection and cultivation can control agricultural productivity, in agricultural policy assessment and to calibrate rates and premiums in crop insurance. Similarly, understanding the trend of crop yield and the insights gained from crop yield predictions can go a long way in helping to address the current global issue of increase in food prices and demand as well as to understand the associated risk of food production by helping farmers to make informed decisions especially on what and where to grow.

We are also not aware of any previous research that has focused on predicting crop yield in east Africa let alone doing so in such an almost holistic manner as we have done in this paper; so, to bridge this research gap, we follow [13] to provide some crop yield forecast in some east African countries. We believe that the results herein will be of extreme importance to east African regional farmers.

The aim of this paper is two folded. First, to forecast the crop yield and secondly to identify cash crops that are capable of producing extremely high yield in some east African countries by modelling the tail region of crop yield data. The remainder of this paper contains data in Section 2, methods in Section 3, results and discussion in Section 4 and conclusions in Section 5.

We use two methods for analyzing the data: time series analysis and fit of heavy tailed distributions. Time series analysis and forecasting is a branch of statistics. Time series forecasting uses models to predict future outcomes based on past observations. With time series visualizations, trends and seasonal patterns could be identified. We could then seek to gain deeper insight as regards to the reason behind these trends. Several time series models have been developed, studied and widely applied in many fields. Box-Jenkins’ auto-regressive integrated moving average (ARIMA) model [14] arguably stands out among others as the most widely used perhaps due to its simplistic application appeal and high precision in modelling. For instance, [15] used the ARIMA model to forecast rice production, consumption, importation, exportation and self-sufficiency in the Benin Republic. [16] used the ARIMA model to forecast the consumption of some livestock products such as eggs, milk, chicken and cow meat to see if the forecast of consumption was on the increase. [17] highlighted that the past century has witnessed significant rise and fall of cocoa production in Nigeria due to diverse institutional and climate changes. They used the ARIMA model to predict cocoa production in Nigeria between 2018 and 2025. Their forecast showed a decreasing trend where cocoa production is expected to fall by more than 20% in 2025 against the 2017 value. [18] used the ARIMA model to forecast maize production in India from 2018 to 2022. The model predicted about 13.76% increase in maize production in India. [19] used the ARIMA model to forecast soybean yield in Zambia. The forecast suggested 23430.3 hectogram / hectare yield increase in 2020 compared to the 2016 figure of 19624 hectogram / hectare. [20] used the ARIMA model to forecast Kharif rice production in West Bengal, India which contributes about 15% of the total paddy in India. [21] used the ARIMA model to forecast sorghum production in South Africa from 2017 to 2020. Their forecast depicted an increasing trend. [22] used the ARIMA model to forecast sugar cane production in Pakistan from 2019 to 2030. Their forecast indicated a significant increase.

Quantifying the tail of the crop yield distribution is vital for managing agricultural production risk and rating crop insurance [23]. The simplest and the most widely used distribution for modelling rare outcomes occurring in the tail region is the power law distribution. Many processes follow the power law over large magnitude of values. Recent examples are the distribution of stock returns [24], income [25, 26], wealth of world billionaires [27], persisters-antibiotic-tolerant cells [28], duration size of unhealthy air pollution events [29], tourism recommendations [30], cumulative coal production [31], agricultural land size [32], rates of wetland loss [18], union size [33], strike size [34] and growth rate of CO_2_ [35]. Popular alternatives to the power law distribution are the lognormal, stretched exponential, and Fréchet distributions.

## 2 Data

Yearly data from 1961 to 2018 on the yield of cash crops like banana, plantain, beans, cassava, coffee, sorghum, potato, sweet potato, maize, rice, sugar cane, wheat, millet and cotton seed from six countries in east Africa (namely, Burundi, Kenya, Somalia, Tanzania, Uganda and Rwanda) were obtained from Food and Agriculture Organization of United Nations-FAO, see http://www.fao.org/faostat/en/#home. The data obtained were yields aggregated at national levels.

**Fig 1:**
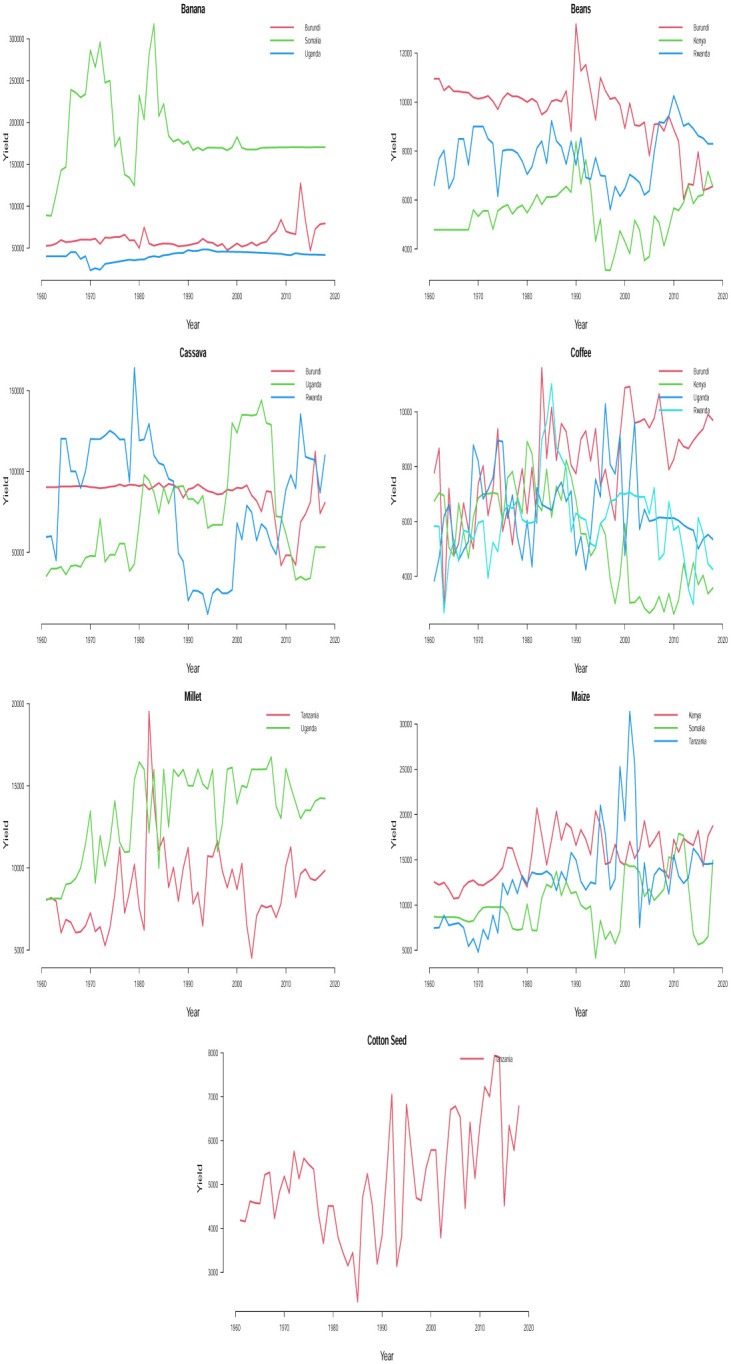
Time series plots for crop yield in different countries.

**Fig 2:**
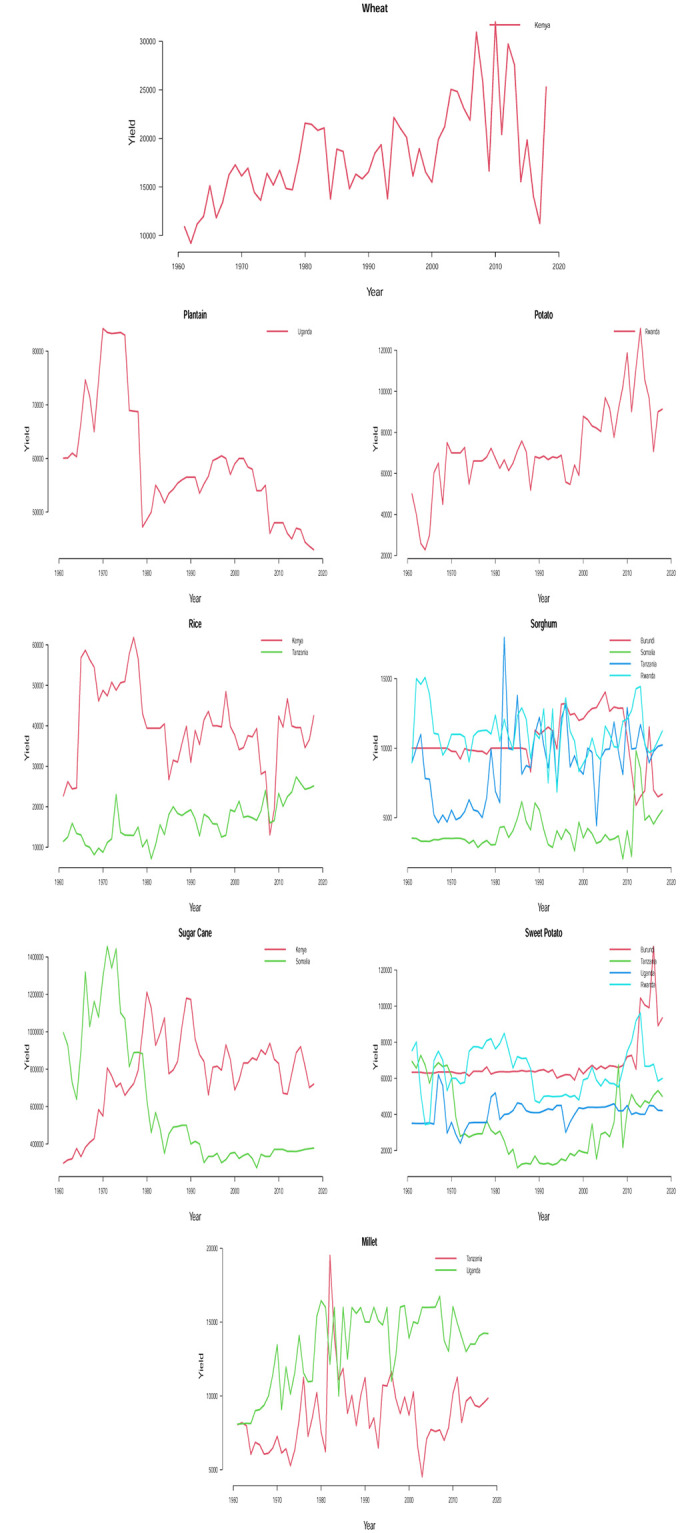
Time series plots for crop yield in different countries.

The time plots of the crops in different countries are shown in Figs 1 and 2. Some sudden changes, particularly big drops and falls could be seen at different times indicating periods of high and low yields. These changes could be as a result of the global economic outlook, environmental/climate changes or even changes in farming practices.

Some descriptive statistics of the data for crops are presented in Table 1. The statistics include the mean, median, standard deviation, minimum, maximum, skewness and kurtosis. The discrepancy between the mean and the median values appears not to be large for almost all the crops across the countries. The mean is larger than the standard deviation for all the crops across the countries. This suggests that the data are underdispersed. Note that underdispersion could be as a result of serial correlation which is typical of time series data. We can remove serial correlation by random variable transformation. But, this may lead to (a) loss of data information and (b) limits us to specific class of models to use. The data exhibit varying degrees of skewness and kurtosis across crops and countries. The lowest (highest) positive skewness of 0.0261 (3.1150) corresponds to maize (sweet potato) in Kenya (Burundi). The lowest (highest) negative skewness of -0.0934 (-2.0220) corresponds to rice (cassava) in Kenya (Burundi). The lowest (highest) positive kurtosis of 0.0410 (13.0916) corresponds to sorghum (banana) in Rwanda (Burundi). The lowest (highest) negative kurtosis of -0.0043 (-1.0478) corresponds to coffee (maize) in Uganda (Kenya). Crop yield skewness has been used to characterize crop yield tendencies. [36] reported that crop yield is positively skewed in the presence of independent, identical and uniform resource availability distribution. Crop yield is negatively skewed whenever the distributions are Gaussian, i.e. skewness depends on asymmetries in resource availabilities, meaning that a negatively skewed yield occurs whenever production is tightly controlled so that the left tails of some resources availabilities distributions are thin [36]. However, in addition to the observable similarities between the mean and the median crop yield values, we notice that for majority of the cases, the skewness and kurtosis values are close to zero, suggesting possible symmetry and mesokurtosis.

**Table 1:** Descriptive measures for the crop yield data sets.

Country	Crop	Min.	1st qu.	Median	Mean	3rd qu.	Max.	Std. dev.	Skewness	Kurtosis
Burundi	Banana	46915	54433	56947	60700	62984	127352	12204.970	3.0704	13.0916
	Beans	6044	9067	10036	9599	10380	13184	1375.755	-0.8491	0.9814
	Cassava	41867	85880	89890	85078	90894	112378	12870.850	-2.0220	4.2142
	Coffee	2687	6961	8251	8100	9379	11598	1769.862	-0.5861	0.1126
	Sorghum	5890	9837	10000	10426	11881	14042	1886.384	-0.3327	-0.1633
	Sweet potato	59048	63302	63837	68215	65848	133015	13063.090	3.1150	10.0345
Kenya	Beans	3127	4783	5556	5413	6122	8382	1034.896	0.0585	0.3111
	Coffee	9212	14918	16839	18293	21078	31991	5003.894	0.7453	0.2387
	Maize	10713	12957	15813	15507	17266	20712	2605.723	0.0261	-1.0478
	Rice	13076	34802	39621	39988	46510	61813	10166.130	-0.0934	-0.0593
	Sugar Cane	297552	689809	808548	774305	885805	1211845	217545.200	-0.4537	-0.0124
	Wheat	9212	14918	16839	18293	21078	31991	5003.894	0.7453	0.2387
Somalia	Banana	88430	169641	170374	185025	198616	317500	46006.370	0.7969	0.7965
	Maize	4149	8173	9758	10065	11765	17901	3029.413	0.5619	-0.1600
	Sorghum	2040	3320	3522	3992	4280	9824	1306.699	2.2982	6.9370
	Sugar Cane	272727	350000	407143	604201	887500	1455975	346839.200	1.0554	-0.2674
Tanzania	Maize	4808	9170	12722	12734	14414	31359	4893.517	1.3077	2.9604
	Millet	4522	7010	8308	8697	9982	19507	2401.514	1.5969	5.1457
	Rice	7143	12826	16286	16396	19172	27382	4933.184	0.2870	-0.7743
	Cotton Seed	2328	4356	5136	5117	5783	7936	1245.132	0.2257	-0.4752
	Sorghum	4423	6554	9151	8832	10100	17963	2717.268	0.4293	0.5903
	Sweet potato	10448	18029	29252	34621	49412	72759	19284.670	0.5541	-1.0485
Uganda	Banana	23298	39412	42070	40891	44927	48333	5636.359	-1.3614	1.613
	Cassava	32973	44771	66988	71711	89971	144083	32354.340	0.7664	-0.5467
	Coffee	3839	5443	6131	6402	7150	10283	1408.237	0.6347	-0.0043
	Millet	8092	11486	14017	13341	15986	16751	2637.066	-0.6120	-0.945
	Plantain	42971	52141	56585	59014	60867	84235	11223.650	0.8573	-0.0373
	Sweet potato	24009	35504	41558	40660	44017	62075	6398.938	0.3221	1.5048
Rwanda	Beans	5606	6980	8020	7858	8522	10258	1037.677	-0.0566	-0.8005
	Cassava	11778	55212	91644	82820	116873	164000	37331.23	-0.2683	-1.0465
	Coffee	2678	5267	5994	6051	6776	11019	1473.642	0.6369	1.6200
	Potato	22821	64313	68656	71994	82871	130600	20435.600	0.2417	0.7926
	Sorghum	6850	10014	11000	11109	12086	15084	1754.313	0.2655	0.0410
	Sweet potato	34388	53682	62550	63978	75039	96163	13269.530	0.0977	-0.4860

**Fig 3:**
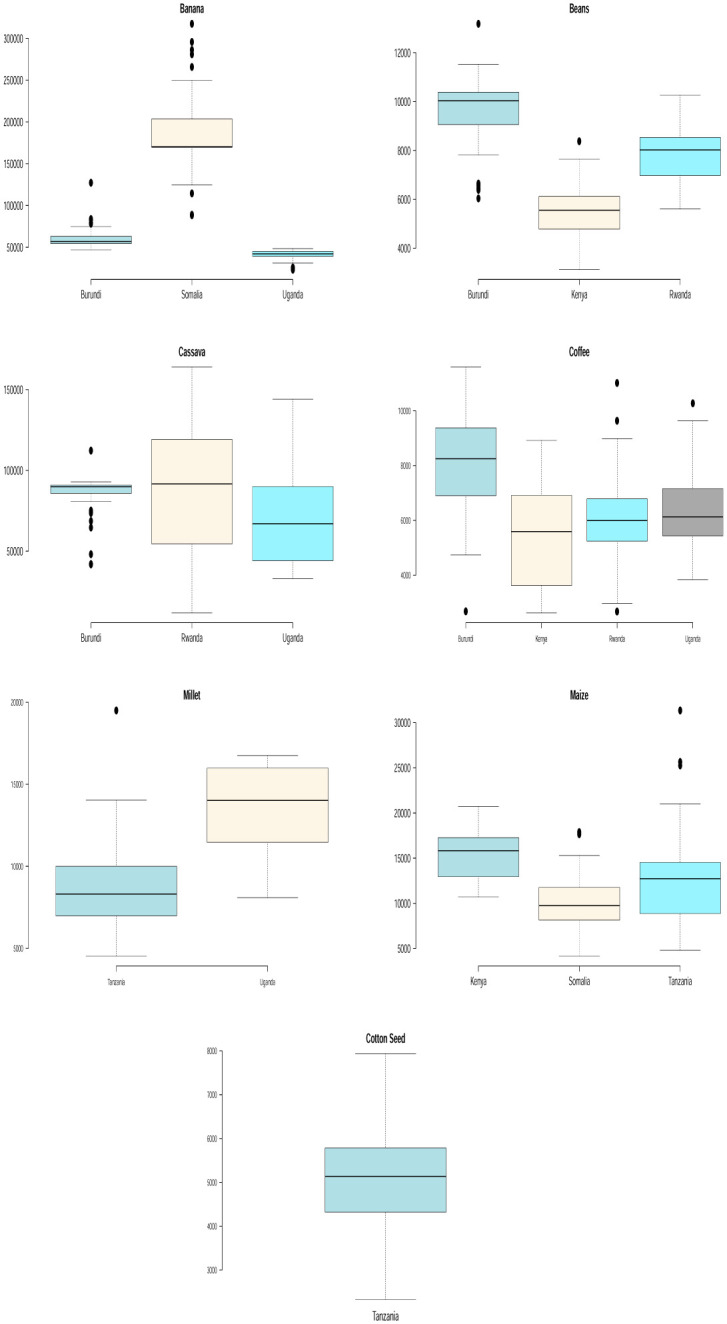
Box plots for crop yield in different countries.

**Fig 4:**
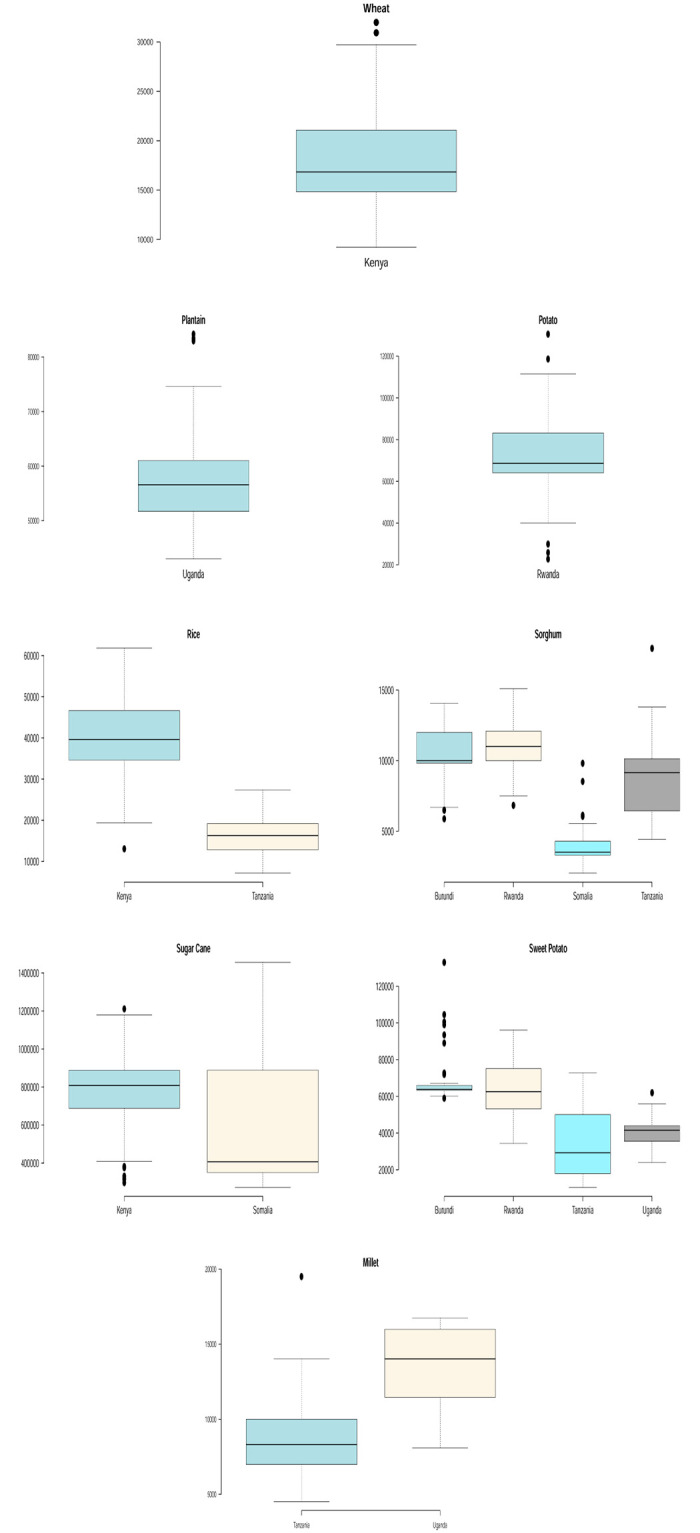
Box plots for crop yield in different countries.

Figs 3 and 4 show boxplots to support the descriptive statistics in Table 1 and to compare the yield performance of some of the crops that are produced in more than one east African country. We see that Somalia recorded the highest banana and sugar cane yields. Burundi recorded the highest beans, coffee and sweet potato yields. Rwanda recorded the highest cassava yield. Kenya recorded the highest rice yield. Tanzania recorded the highest sorghum, maize and millet yields. Also, evident enough in Figs 3 and 4 are the presence of extreme (high and low) yields for some of the crops which are indicated by observations lying outside of the whiskers in the box plots. The power law distribution discussed later is especially useful for modelling unusually high yields.

We tested heavytailedness of the each data set using [37]’s test based on Kolmogorov-Smirnov statistic corrected for correlation [38]. The *p*–values of this test for banana, beans, cassava, coffee, sorghum and sweet potato in Burundi were 0.182, 0.0664, 0.151, 0.102, 0.156 and 0.115, respectively. The *p*–values for the crops in Kenya were 0.162, 0.171, 0.059, 0.120, 0.166 and 0.145. The *p*–values for the crops in Somalia were 0.167, 0.157, 0.098 and 0.115. The *p*–values for the crops in Tanzania were 0.168, 0.112, 0.095, 0.068, 0.096 and 0.125. The *p*–values for the crops in Uganda were 0.177, 0.114, 0.087, 0.171, 0.105 and 0.077. The *p*–values for the crops in Rwanda were 0.075, 0.169, 0.068, 0.098, 0.061 and 0.158. The *p*–values reported show that there is no significant evidence against the fact that each data has a heavy tail. Hence, unusually high yields can be modeled by heavy tailed distributions as done in Section 4.

## 3 Methods

### 3.1 Time series analysis of crop yields

One possible technique for time series analysis is to assume that the overall mean is either constantly increasing or constantly decreasing with respect to time. In this case, the fit of a sloping line might be appropriate for the time series. This type of line is typically referred to as a linear trend model or a trend-line model and it is a special case of a simple linear regression model with time index *t* as the only predictor variable, i.e. *t* = 1, 2, 3, …. The estimated trend line is the line that minimizes the sum of the squared vertical deviations from the data. Trend lines serve as important visual aids. However, they often perform poorly in forecasting beyond the historical data. In practice, majority of the time series data that arise in different areas cannot be described by some straight lines because their trends often undergo evolution. Given the past observations, the trend-line model attempts to find the intercept and slope that give the best average fit to the data. Unfortunately, the deviation of the linear trend model from the data is usually greatest at the end of the time series where the forecasting starts. Therefore, in time series analysis and forecasting, the important question ‘what is the appropriate model?’ can first be addressed by visually inspecting the time series data for any constantly changing trend or randomly changing trend. Based on Figs 1 and 2, we see that assuming a steady upward or downward linear trend for any of the crop yield data is apparently illogical and out of place because a randomly changing trend is overwhelmingly evident for all the time series data. To model the nonlinear trend in all the time series, we may need to regress the time series on second or higher order terms of *t* and this may require some trial and errors which may possibly lead to some overestimated or underestimated models. To circumvent the issue of model selection, we consider the most reliable models for nonlinear trends in time series and they are referred to as stochastic time-series models. Examples of such models are the one proposed by [14] which involve straightforward laid down iterative procedures for model fitting unlike the nonlinear regression method mentioned earlier.

In this section, we carry out a time series analysis to study the yield pattern of crops over a specified period of time. We need to isolate first the impact of trends (the overall pattern in the series) and second the impact of random disturbances (the vigorous wiggles in the series). The impact of trends could be due to planting strategies and techniques, advanced mechanized farming, farm management, irrigation, the use of fertilizers and genetically improved seedlings/crops. The impact of random disturbances could be due to pandemics, crop disease outbreaks, wars, recessions, environmental degradations (for example, erosion) and extreme weather conditions such as droughts and floods.

Let *x*_*t*_ denote the observed yield of a crop at time *t*. Suppose we denote all the observed information up to time *t* by $I_{t}$. We are interested in forecasting *x*_*t*_. We can specify the forecast as $x_{t}|I_{t}$ or more specifically as ${\hat{x}}_{t+h}|t$. The forecast of *x*_*t*+*h*_ given all previous observations up to time *t* (*x*_1_, *x*_2_, …, *x*_*t*_) is known as the *h*–step forecast. The *h*–step forecasting method can be easily implemented through the famous Box-Jenkins autoregressive integrated moving average (ARIMA) modelling framework. ARIMA models are used for trend analysis and forecasting. The ARIMA (*p*, *d*, *q*) model is defined by

$$\begin{array}{c} {}[1-\sum_{i=1}^{p}\phi_{i}B^{i}]{\left(1-B\right)}^{d}x_{t}=c+[1+\sum_{j=1}^{q}\theta_{j}B^{j}]\xi_{t}, \end{array}$$

where *ϕ*’s are the autoregressive (AR) parts of the model, *θ*’s are the moving average (MA) parts of the model, *d* is the order of difference, *B* is known as the backshift operator, *c* is a constant which is equal to *μ*(1 − *ϕ*_1_ − ⋯ − *ϕ*_*p*_), *μ* is the mean of the *d*th differenced series (1 − *B*)^*d*^*x*_*t*_ and *ξ*_*t*_ is white noise. *ξ*_*t*_ are generally assumed to be independent, identically distributed variables sampled from a normal distribution with zero mean. In ARIMA modelling, we make the following assumptions about the time series: there are no seasonality or cyclical trends, there are no outliers, and that the variation about the mean is consistent. After fitting the ARIMA model, we can check the model adequacy viz-a-viz a popular portmanteau test called Ljung-Box test by simply testing whether the residuals from the fitted model are white noise. For Ljung–Box test, we test the hypothesis *H*_0_: *ρ*_*k*_ = 0 versus *H*_1_: *ρ*_*k*_ ≠ 0. The test statistic of Ljung-Box test is

$$\begin{array}{c} Q^{⋆}=n\left(n+2\right)\sum_{k=1}^{h}\frac{{\hat{\rho }}_{k}^{2}}{\left(n-k\right)}, \end{array}$$

where *n* is the sample size, ${\hat{\rho }}_{k}$ is the sample autocorrelation at lag *k*, and *h* is the number of lags being tested. Under *H*_0_, the statistic *Q*^⋆^ is asymptotically chi-square distributed with *h* degrees of freedom. At *α* significance level, the critical region for rejecting the hypothesis of randomness is $Q^{⋆}>\chi_{1-\alpha ,h}^{2}$, where $\chi_{1-\alpha ,h}^{2}$ denotes the (1 − *α*)th quantile of the chi-squared distribution with *h* degrees of freedom.

A detailed discussion of Box-Jenkins ARIMA (*p*, *d*, *q*) model could be read from [39] and [40]. In Figs 1 and 2, we find some evidence of changing variance in some of the series. Each series appears clearly non-stationary as the series wanders up and down. Before proceeding with the data analysis, we ensured that the variance for each series is stabilized by the Box-Cox transformation [41].

The Box Cox transformation involves an exponent, λ ∈ [−5, 5]. In this paper, all values of λ are considered but the optimal value for each data is applied. The optimal value of λ is the one that gives the best approximation of the Gaussian distribution. The transformation of *x*_*t*_ has the form:

$$\begin{array}{c} x_{t}\left(\lambda \right)=\{\begin{array}{cc} \frac{x_{t}^{\lambda }-1}{\lambda }, & \text{if}\;\lambda \neq 0,\\ \text{ln}\;(x_{t}), & \text{if}\;\lambda =0. \end{array} \end{array}$$ (1)

The formula in (1) is not as simple as it appears because testing for all possible values one by one is unnecessarily time consuming. However, most software packages include an option for a Box-Cox transformation. In this paper, we used the ${}^{\prime }\text{auto}.{\text{arima}}^{\prime }$ function in the ${}^{\prime }{\text{forecast}}^{\prime }$ package in the R (R Core Team, 2022) software to fit the ARIMA (*p*, *d*, *q*) models. Setting the ${}^{\prime }{\text{lambda}}^{\prime }$ argument to ${}^{\prime }{\text{auto}}^{\prime }$ allows a transformation to be automatically selected and implemented using the Box-Cox method. The routinely transformed data are then coerced into stationarity by implementing first or second order differences whenever there is any need to do so before estimating the appropriate model.

Each coerced series was tested for stationarity using [42]’s test. The null hypothesis was that the series is stationary. The *p*–values for banana, beans, cassava, coffee, sorghum and sweet potato in Burundi were 0.085, 0.085, 0.089, 0.095, 0.075 add 0.083, respectively. The *p*–values for the crops in Kenya were 0.057, 0.081, 0.051, 0.069, 0.086 and 0.056. The *p*–values for the crops in Somalia were 0.098, 0.078, 0.089 and 0.083. The *p*–values for the crops in Tanzania were 0.067, 0.078, 0.082, 0.083, 0.081 and 0.052. The *p*–values for the crops in Uganda were 0.094, 0.086, 0.095, 0.051, 0.092 and 0.090. The *p*–values for the crops in Rwanda were 0.098, 0.099, 0.053, 0.073, 0.090 and 0.080.

### 3.2 Analysis of the maximum crop yields

Suppose we denote the crop yield random variable by *X* with realizations *x*_*i*_, *i* = 1, 2, …, *n*, where *n* represents the number of observations. For the convenience of fitting distributions to the available data, we assume that the *x*_*i*_ are random. The assumption of independence is not technically correct as the data are actually serially correlated. But ignoring dependence in a data set and treating the data as being independent has no effect on parameter estimates, it only affects standard errors (see, for example, [43]). Hence, the results presented later on the fit of heavy tailed distributions are correct as accuracy of estimation is not taken into account.

The probability density functions (PDFs) of the fitted heavy tailed distributions are

1. The power law distribution also known as Pareto distribution of type I [44] specified by the PDF
  $$\begin{array}{c} f\left(x\right)=\frac{\alpha -1}{x_{\text{min}}}(\frac{x}{x_{\text{min}}}{)}^{-\alpha } \end{array}$$
  for *x* ≥ *x*_min_ > 0, where *x*_min_ is the lower bound and *α* > 0 is the exponent. At or above *x*_min_, the distribution exhibits properties of a power law distribution.
2. The lognormal distribution specified by the PDF
  $$\begin{array}{c} f\left(x\right)=\frac{1}{bx\sqrt{2\pi }}\;\text{exp}\;[-\frac{{\left(\text{ln}\;x-a\right)}^{2}}{2b^{2}}] \end{array}$$
  for *x* > 0, where −∞ < *a* < ∞ and *b* > 0 are the location and scale parameters, respectively.
3. The stretched exponential distribution specified by the PDF
  $$\begin{array}{c} f\left(x\right)=\frac{b}{a}{\left(\frac{x}{a}\right)}^{b-1}\;\text{exp}\;[-{\left(\frac{x}{a}\right)}^{b}] \end{array}$$
  for *x* > 0, where *a* > 0 is the scale parameter and *b* > 0 is the shape parameter.
4. Fréchet distribution [45] specified by the PDF
  $$\begin{array}{c} f\left(x\right)=ba^{b}x^{-1-b}\;\text{exp}\;[-{\left(\frac{x}{a}\right)}^{-b}] \end{array}$$
  for *x* > 0, where *a* > 0 is the scale parameter and *b* > 0 is the shape parameter.

We estimated the parameters of all the distributions by the method of maximum likelihood through the optim routine in R [46]. We estimated *x*_min_ in the power law distribution by following the method in [47]. That is, we chose *x*_min_ that minimized

$$\begin{array}{c} \text{KS}=\underset{x\geq x_{\text{min}}}{\text{max}}|F_{n}\left(x\right)-\hat{F}\left(x\right)|, \end{array}$$

where *F*_*n*_(*x*) and $\hat{F}\left(x\right)$ denote, respectively, the empirical and fitted power law distribution functions for *x* ≥ *x*_min_.

We have used the method of maximum likelihood because of its popularity. There are other methods for estimation; in particular, for estimating *α* of the power law distribution. Some of these estimators include the rank estimator due to [48], [49]’s estimator and the median estimator due to [50].

Note that each of the four distributions has two free parameters. So, no one distribution is more flexible than the others in terms of the number of parameters. Unlike the power law distribution, the lognormal, Fréchet and stretched exponential distributions model the entire data. We can compare their fits by the following goodness-of-fit measures:

1. Bayes information criterion (BIC) due to [51] defined by
  $$\begin{array}{c} \text{BIC}=-2\hat{L}+k\;\text{ln}\;\left(n\right), \end{array}$$
2. Akaike information criterion with a correction (AICc) due to [52] defined by
  $$\begin{array}{c} \text{AICc}=\text{AIC}+\frac{2k(k+1)}{n-k-1}, \end{array}$$

where $\hat{L}$ and *k* denote, respectively, the maximized log likelihood value and the number of unknown parameters.

We can also compare all of the fitted distributions through the Kolmogorov–Smirnov test. Its statistic is given by

$$\begin{array}{c} \text{KS}=\underset{x\in \text{Data}}{\text{max}}|F_{n}\left(x\right)-\hat{F}\left(x\right)|, \end{array}$$

which was corrected as in [38] to account for correlation in the data. The larger the value of the corresponding KS *p*–value the better the fitted distribution. We require the *p*–value of the Kolmogorov–Smirnov test to be greater than 0.05 to conclude that the distribution is a reasonable model for the data. A *p*–value less than 0.05 suggests an absolute rejection of the distribution as a candidate for the data. However, one major drawback of the Kolmogorov–Smirnov *p*–value is that it depends on fixed parameters, hence it does not reflect sampling variability. We can calculate more conservative *p*–values by a bootstrapping method in [47]. We implemented this method by using 5000 bootstrap replications to obtain the final *p*–value for the Kolmogorov–Smirnov test. In this paper, we shall use the non-bootstrapped KS *p*–value to verify the plausibility of each distribution as a candidate model for data. We use the bootstrapped KS *p*–value to discriminate among competing distributions and to generalize our findings.

Vuong test [53] can be used to discriminate between two non-nested models by testing the null hypothesis that the models provide indistinguishable fits for the same data. Suppose we denote the probabilities for models 1 and 2 by $P(\text{x}|\hat{\Theta_{1}})$ and $P(\text{x}|\hat{\Theta_{2}})$, respectively, where $\hat{\Theta_{1}}$ and $\hat{\Theta_{2}}$ denote the parameter estimates for models 1 and 2, respectively. Let $\text{d}=\text{ln}\;P(\text{x}|\hat{\Theta_{1}})-\text{ln}\;P(\text{x}|\hat{\Theta_{2}})$. The test statistic for Voung’s test is $\Lambda =\frac{\sqrt{n}\bar{d}}{s_{d}}$, where $\bar{d}$ and *s*_*d*_ denote the mean and standard deviation of **d**, respectively. A large, positive test statistic value provides evidence that model 1 is superior to model 2. A large, negative test statistic value gives evidence that model 2 is superior to model 1. Under the null hypothesis that the models are inseparable, the test statistic Λ is asymptotically standard normal distributed. Two finite sample corrections of Vuong’s test are sometimes considered based on the AIC and BIC penalty terms, depending on the complexity of the two models. However, these corrections sometimes generate conflicting conclusions.

## 4 Results and discussion

**Fig 5:**
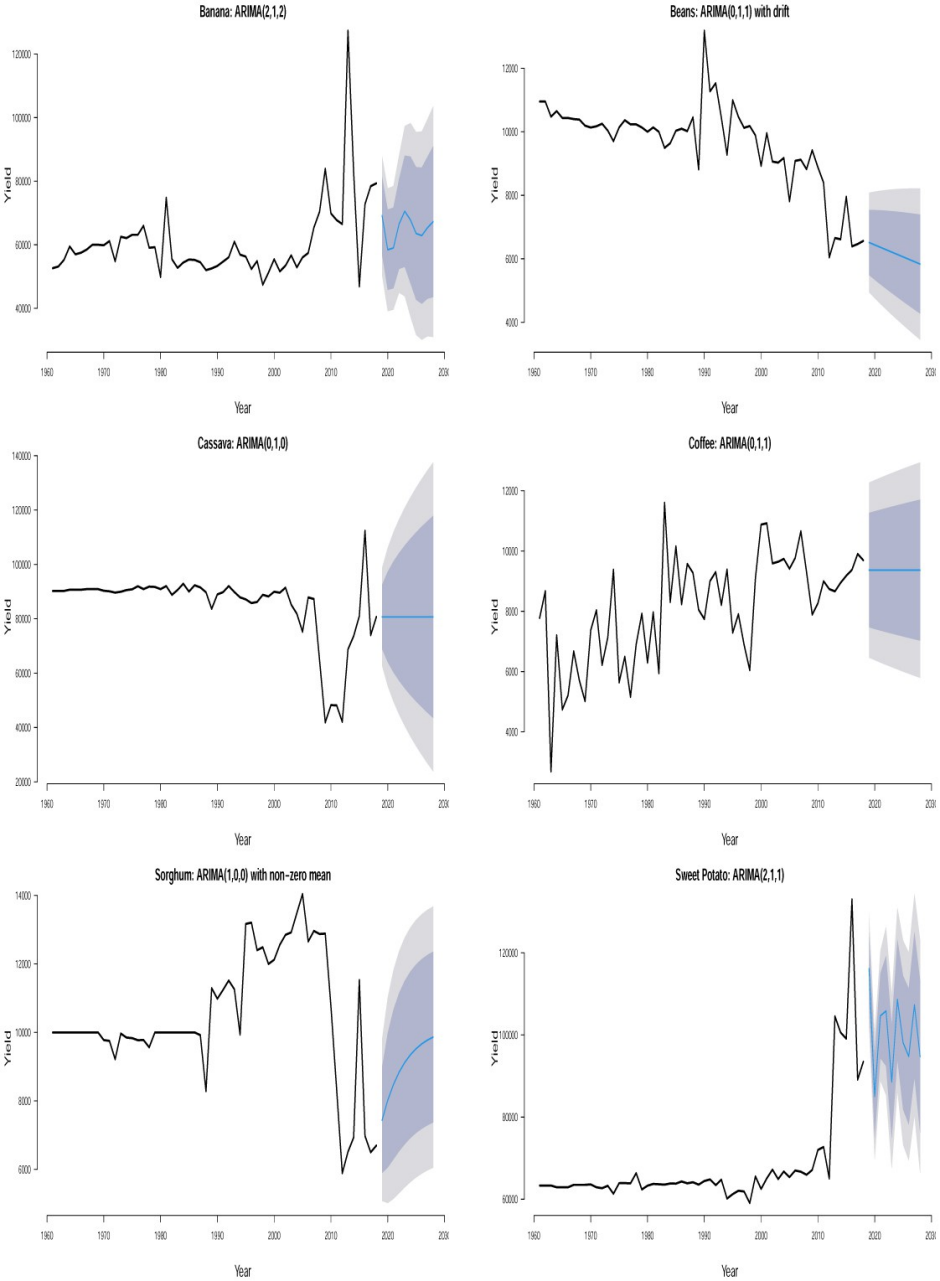
Time series plots and 10 years yield forecast with fitted ARIMA models showing 80% and 95% confidence bands for crops in Burundi.

**Fig 6:**
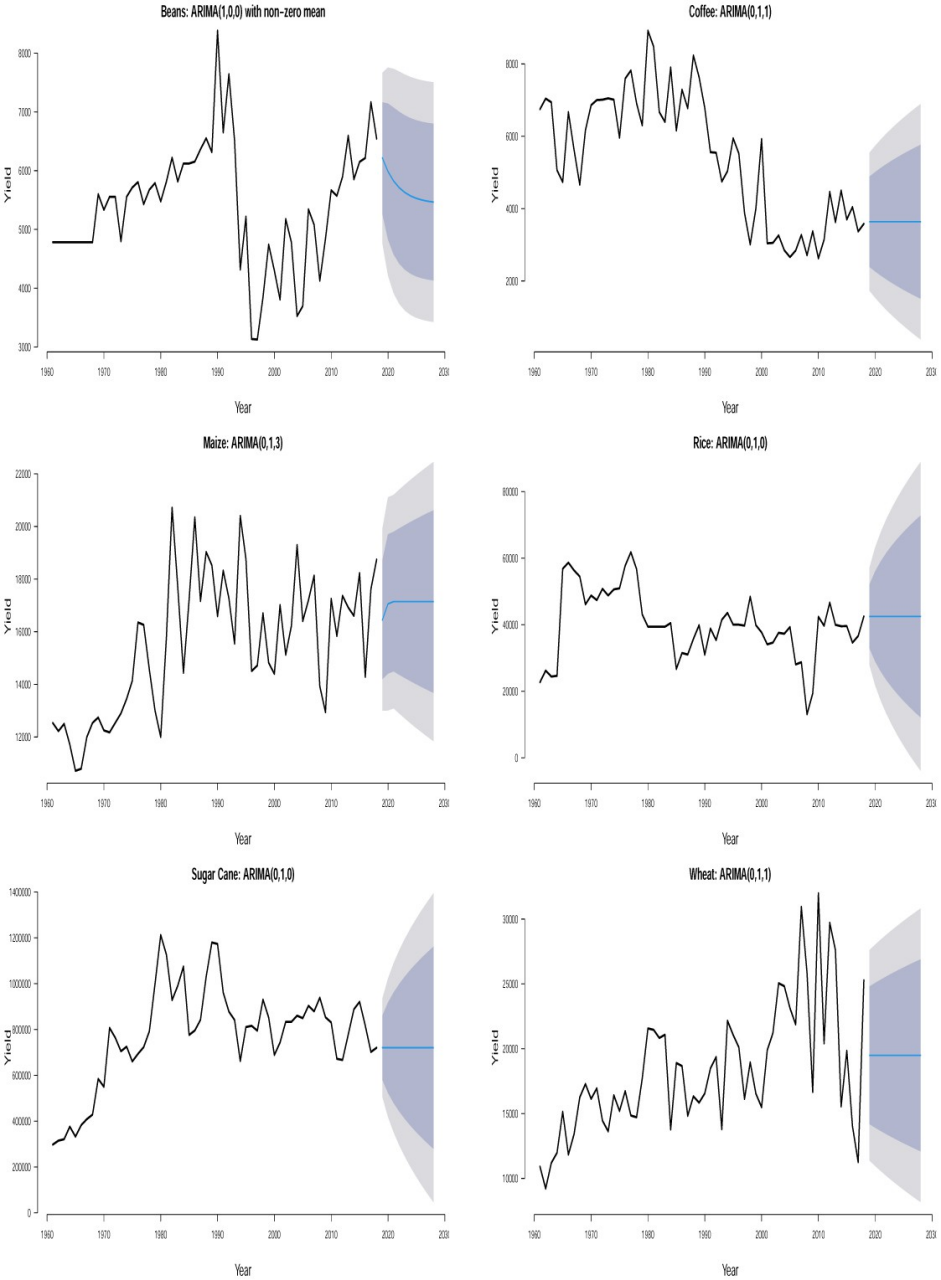
Time series plots along with 10 years yield forecast for the fitted ARIMA models showing 80% and 95% prediction confidence bands for crops in Kenya.

**Fig 7:**
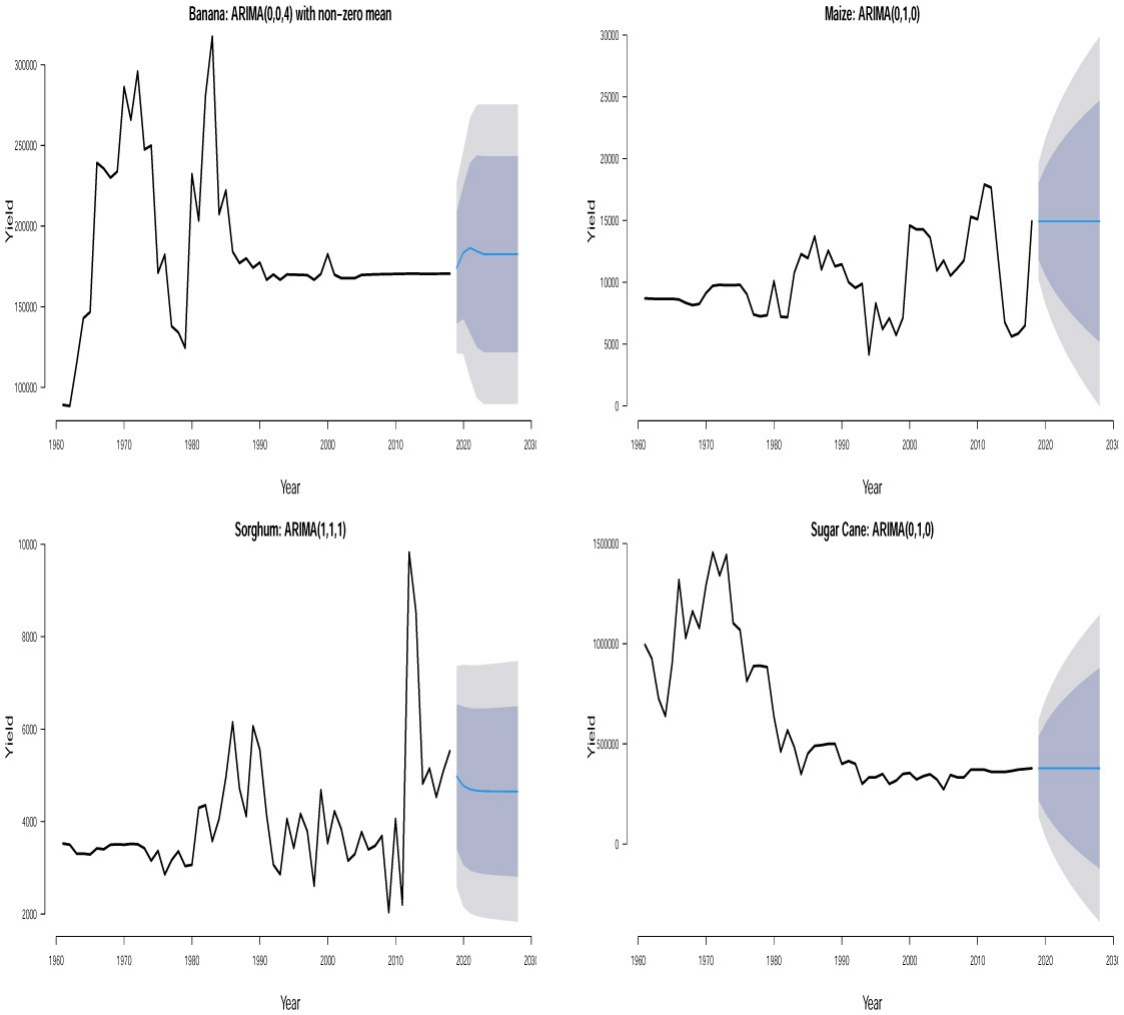
Time series plots along with 10 years yield forecast for the fitted ARIMA models showing 80% and 95% prediction confidence bands for crops in Somalia.

**Fig 8:**
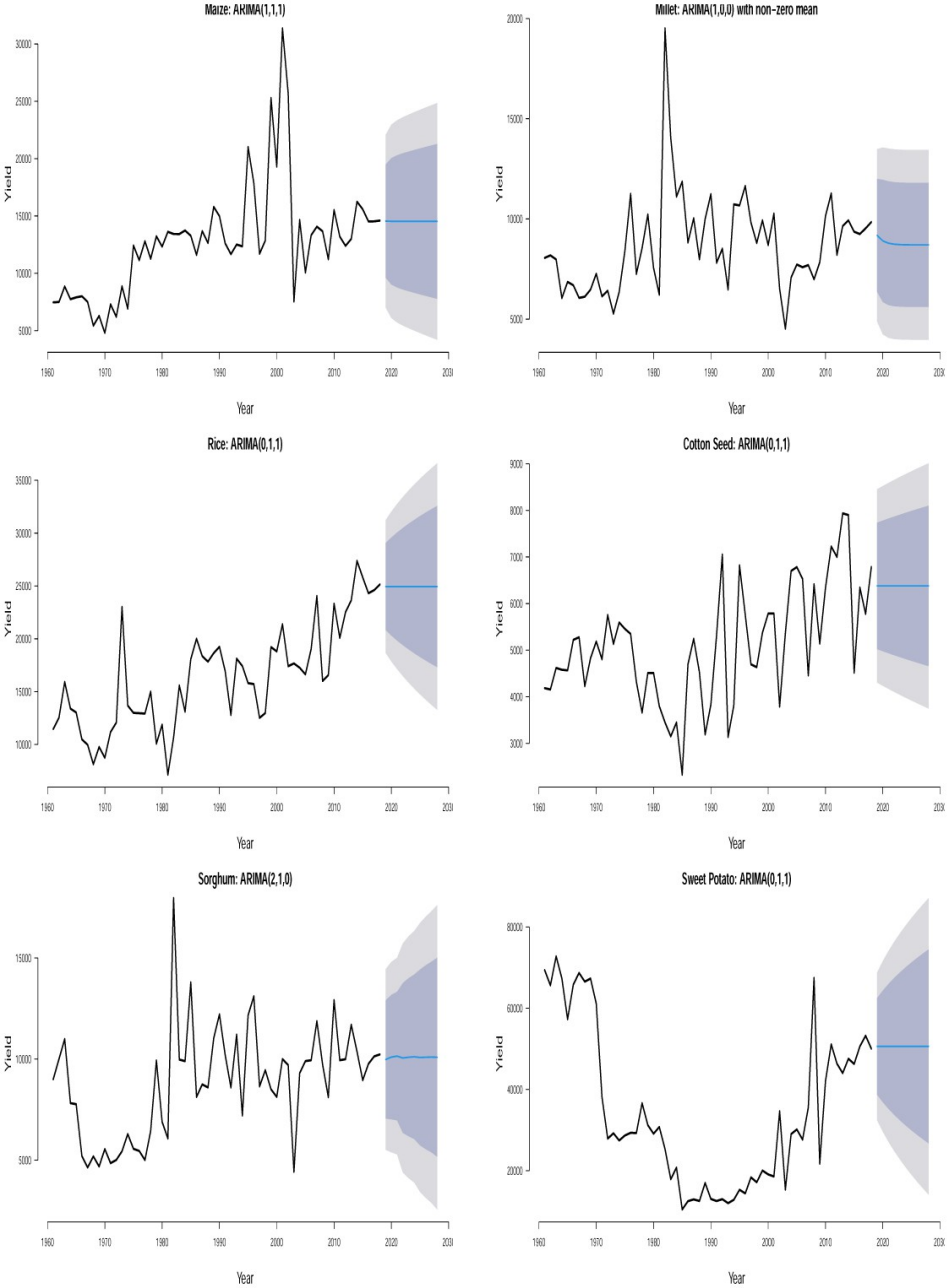
Time series plots along with 10 years yield forecast for the fitted ARIMA models showing 80% and 95% prediction confidence bands for crops in Tanzania.

**Fig 9:**
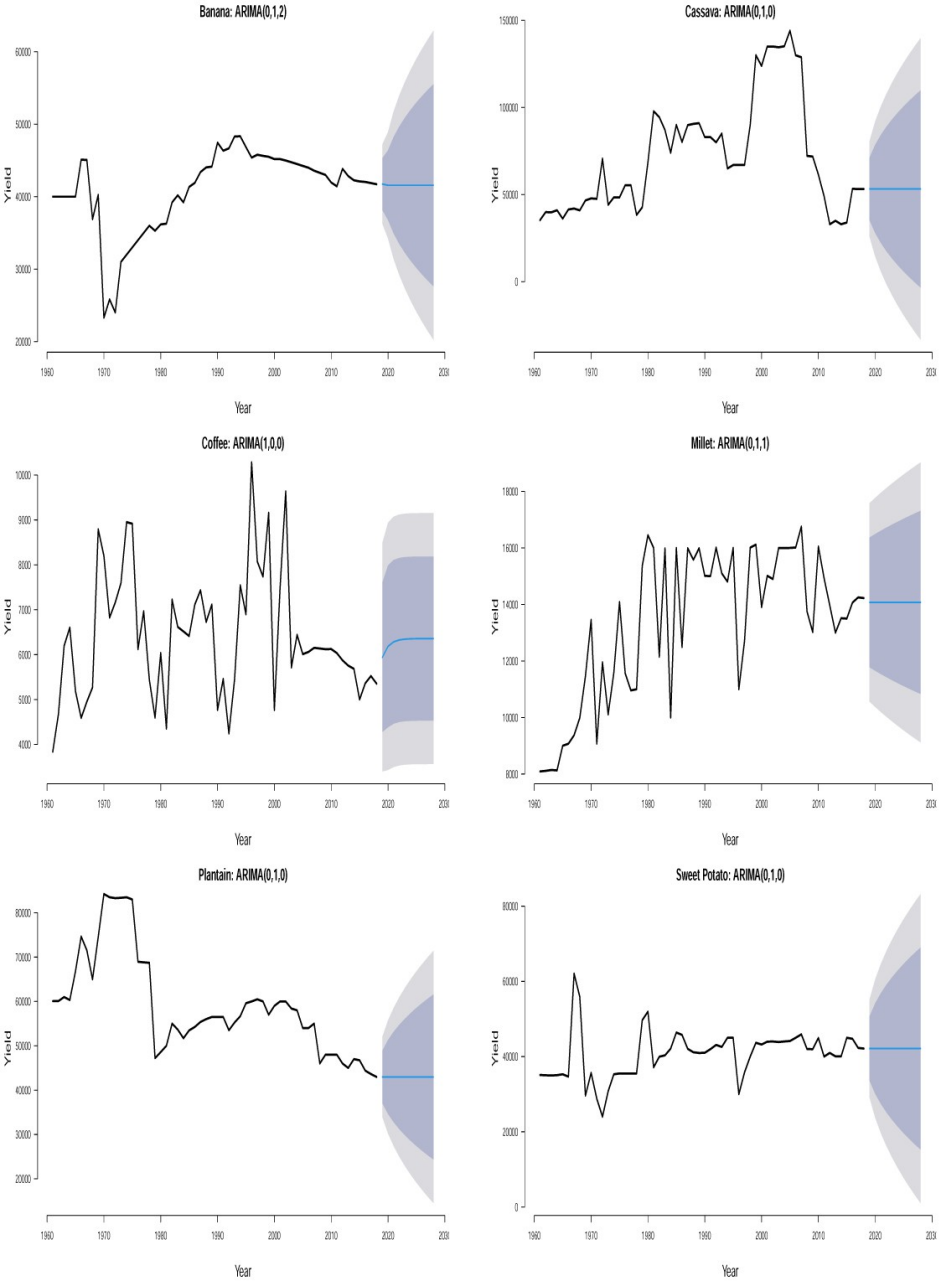
Time series plots along with 10 years yield forecast for the fitted ARIMA models showing 80% and 95% prediction confidence bands for crops in Uganda.

**Fig 10:**
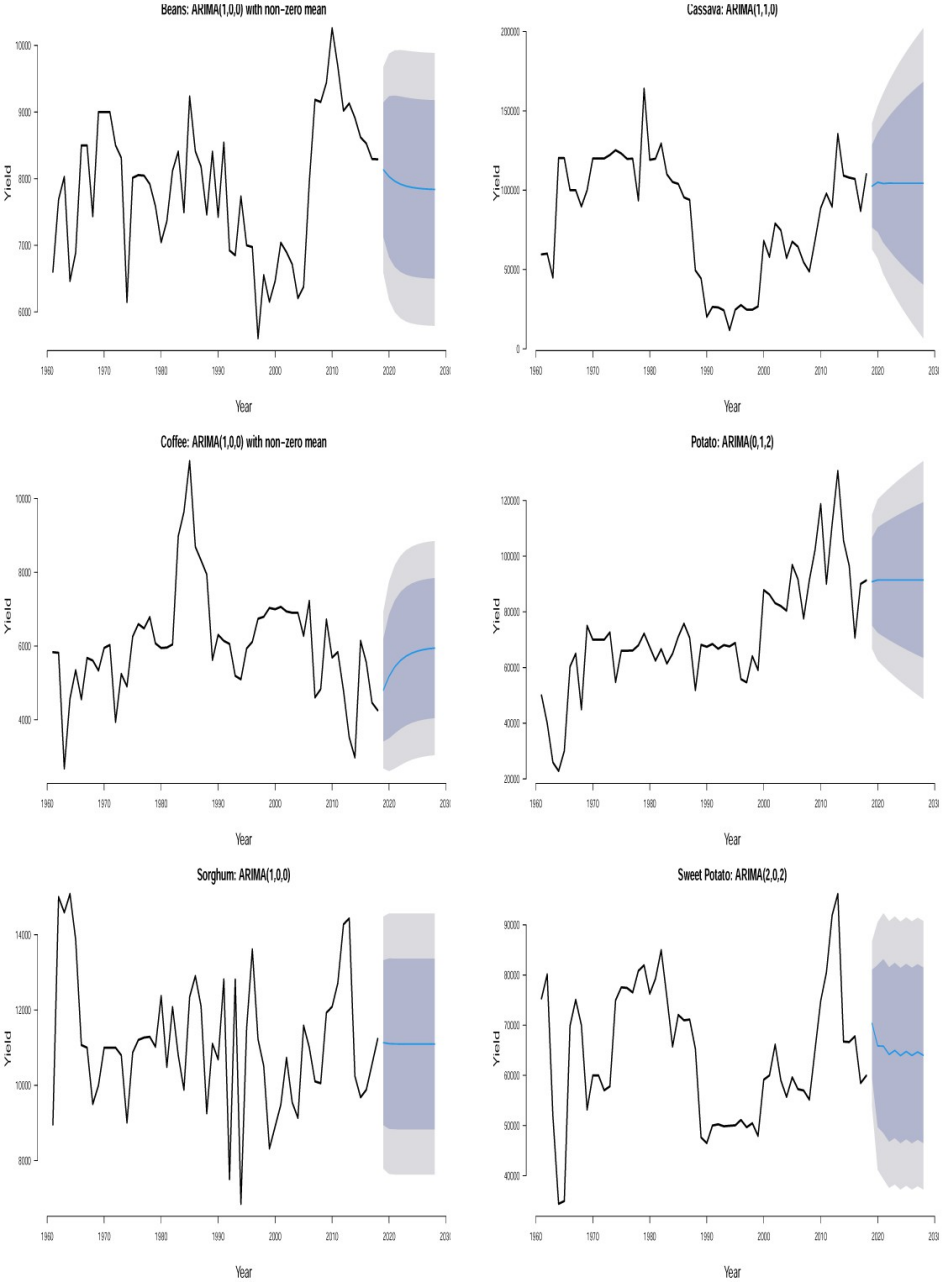
Time series plots along with 10 years yield forecast for the fitted ARIMA models showing 80% and 95% prediction confidence bands for crops in Rwanda.

Ljung–Box *p*–values in Table 2 are > 0.05 suggesting that the residuals of the fitted ARIMA models are not statistically significant from white noise at 0.05 significance level for all the crops except for plantain in Uganda which is not statistically significant from white noise at 0.01 significance level. All of the fitted models are suitable for prediction based on the residual analysis. From the 10 years (2019–2028) point forecast (solid blue lines) of the fitted ARIMA models in Figs 5 to 10, we observe the following for Burundi: an initial sharp drop in 2019 followed by an upward swing of yield for banana; a sharp increase in 2019 followed by increasing oscillations of yield for sweet potato; the yield for sorghum shows a quick increase from 2019 to 2028; the yield for beans shows an immediate decline from 2019 to 2028; neither cassava nor coffee indicate any increasing or decreasing pattern from 2019 to 2028. In Kenya, we observe the following: the yield for beans shows a continuous decline from 2019 to 2028; neither upward nor downward yield trend is evident for coffee, rice, wheat and sugar cane from 2019 to 2028; the yield of maize shows a sharp drop in 2019 followed by an increase and then a stable trend. In Somalia, we observe the following: the yield for maize or sugar cane does not indicate any pattern; the yield for banana shows an initial moderate increase in 2019 followed by a period of no trend up to 2028; the yield for sorghum first experienced a sharp drop in 2019 followed by a stable period of no trend up to 2028. In Tanzania, we observe the following: no significant trend could be identified for maize, rice, sweet potato and cotton seed for the entire forecast period; millet is characterized by a slight yield decrease in 2019 followed by a period of no significant trend up to 2028. In Uganda, we observe the following: the forecast for banana, cassava, millet, plantain and sweet potato did not show any significant trend from 2019 to 2028; the yield for coffee shows a slight increase in 2019 followed by a period of neither increase nor decrease. In Rwanda, we observe the following: the yield for beans shows a persistent decline from 2019 to 2028; the yield for sweet potato shows initial jump followed by a slow decline; coffee indicated an upward trend tendency from 2019 to 2028; cassava, potato and sorghum did not indicate any significant trend.

**Table 2:** Ljung–Box test statistic (*Q*^⋆^), its degree of freedom and its *p*–value for the fitted ARIMA models at lag 10 (i.e. *h* = 10).

Country	Crop	Fitted ARIMA (*p*, *d*, *q*) ^a^	*Q* ^⋆^	df	*p*–value
Burundi	Banana	ARIMA(2,1,2)	1.5746	6	0.9544
	Beans	ARIMA(0,1,1) with drift	5.9108	8	0.6572
	Cassava	ARIMA(0,1,0)	12.0820	10	0.2796
	Coffee	ARIMA(0,1,1)	8.3159	9	0.5027
	Sorghum	ARIMA(1,0,0) with non-zero mean	8.9597	8	0.3457
	Sweet potato	ARIMA(2,1,1)	7.8161	7	0.3491
Kenya	Beans	ARIMA(1,0,0)	12.0780	8	0.1478
	Coffee	ARIMA(0,1,1)	13.2300	9	0.1525
	Maize	ARIMA(0,1,3)	3.4473	7	0.8408
	Rice	ARIMA(0,1,0)	10.4230	10	0.4042
	Sugar Cane	ARIMA(0,1,0)	17.9800	10	0.0553
	Wheat	ARIMA(0,1,1)	13.9030	9	0.1258
Somalia	Banana	ARIMA(0,0,4) with non-zero mean	4.1658	5	0.5258
	Maize	ARIMA(0,1,0)	15.1300	10	0.1274
	Sorghum	ARIMA(1,1,1)	3.8376	8	0.8715
	Sugar Cane	ARIMA(0,1,0)	13.8200	10	0.1814
Tanzania	Maize	ARIMA(1,1,1)	11.8720	8	0.1570
	Millet	ARIMA(1,0,0) with non-zero mean	6.2974	8	0.6140
	Rice	ARIMA(0,1,1)	7.7560	9	0.5589
	Cotton Seed	ARIMA(0,1,1)	18.8250	9	0.0267
	Sorghum	ARIMA(2,1,0)	4.2521	8	0.8337
	Sweet potato	ARIMA(0,1,1)	12.8150	9	0.1711
Uganda	Banana	ARIMA(0,1,2)	3.8909	8	0.8668
	Cassava	ARIMA(0,1,0)	12.1600	10	0.2745
	Coffee	ARIMA(1,0,0) with non-zero mean	15.3660	8	0.0524
	Millet	ARIMA(0,1,1)	6.1006	9	0.7298
	Plantain	ARIMA(0,1,0)	19.0490	10	0.0397
	Sweet potato	ARIMA(0,1,0)	17.7340	10	0.0596
Rwanda	Beans	ARIMA(1,0,0) with non-zero mean	7.9098	8	0.4423
	Cassava	ARIMA(1,1,0)	2.3680	9	0.9842
	Coffee	ARIMA(1,0,0) with non-zero mean	3.5770	8	0.8931
	Potato	ARIMA(0,1,2)	4.4913	8	0.8103
	Sorghum	ARIMA(1,0,0) with non-zero mean	4.4304	8	0.8164
	Sweet potato	ARIMA(2,0,2) with non-zero mean	2.4531	5	0.7835

^*a*^Due to space constraints, we omit the coefficients of the fitted ARIMA models; interested readers can obtain them from the authors upon reasonable request.

The changes observed in Figs 5 to 10 are consistent with findings in the literature. [54] established that both intra- and interseasonal changes in temperature and precipitation influence cereal yields in Tanzania. [55] reported that climate change will reduce mean yields in Africa by 17% for wheat, 5% for maize, 15% for sorghum and 10% for millet. No mean change in yield for rice was detected. Using data from the northern Tanzanian highlands, [56] demonstrated that increasing night time temperature is the most significant climatic variable responsible for diminishing coffea arabica yields between 1961 and 2012. According to [57], annual food crops in the Kilimanjaro region of Tanzania were particularly sensitive to the drought and maize and beans yields were lower than perennial crops during the years of drought. Through a simulation study, [58] predicted climate change in east Africa and found its negative impact on crop production in that region. They projected that the crop output decrease will lie between 1.2% and 4.5%. [59] identified soil erosion by water as one of the major causes of land degradation and dwindling agricultural produce in Africa resulting in an estimated yearly crop yield loss of about 280 million tons. [60] provided evidence to suggest that climate change severely impacted rice production in Rwanda. [61] produced evidence to suggest that temperature increases lead to decline in maize and cassava crops for Tanzania, Malawi, Zambia and South Africa. [62] observed that the yields for maize, sorghum or millet fluctuated at a decreasing trend in the Kongwa district of Tanzania. According to [63], increased temperatures in Kenya due to climate change have a general tendency to reduce rice yields. [64] showed that the impacts of projected changes in climate on maize production areas are the reduction in the suitability of the crop, especially around central and western Tanzania, mid-northern and western Uganda, and parts of western Kenya by 20–40%, and patches of east Africa will experience a reduction as high as 40–60%, especially in northern Uganda, and western Kenya. According to [65], maize production in southern highlands of Tanzania has decreased during the past two decades, since the year 2000. According to [66], climate change has induced a devastating effect on agricultural production in Somalia leading to crop yield to decline including sorghum.

Tables 3 and 4 give the BIC, AICc and the KS *p*–values of the fitted distributions. The BIC and AICc values for the power law distribution are smaller than those for the remaining distributions. The KS *p*–value > 0.05 in all the cases except for Millet in Uganda indicating that the power law is not a plausible distribution in this case. We cannot compare the values of the goodness-of-fit measures of the power law distribution with those of the other distributions because the power law distribution fits only the tails whereas the lognormal, Fréchet, and the stretched exponential distributions fit the entire data. Thus, we can only compare the BIC and AICc values of the lognormal, Fréchet, and the stretched exponential distributions. Based on the KS *p*–value, we can observe that the lognormal distribution could be a plausible distribution for banana and coffee in Burundi, all the crops in Kenya except for sugar cane, maize and sorghum in Somalia, all the crops in Tanzania, all but banana in Uganda and all but cassava in Rwanda. Fréchet distribution appears to be a plausible distribution for banana in Burundi, maize and wheat in Kenya, maize and sorghum in Somalia, all except for maize and sorghum in Tanzania and cassava, coffee and plantain in Uganda and all except for cassava and potato in Rwanda. The stretched exponential distribution appears to be a plausible distribution for beans and coffee in Burundi, all the crops in Kenya, maize in Somalia, all the crops in Tanzania, all except for plantain in Uganda and all the crops in Rwanda.

**Table 3:** AIC, BIC, AICc and KS *p*–values (as defined in Section 3) for the fitted distributions.

Country—Crop	Power law			Lognormal			Fréchet			Stretched exponential		
	BIC	AICc	*p*–value	BIC	AICc	*p*–value	BIC	AICc	*p*–value	BIC	AICc	*p*–value
Burundi—Banana	723.717	719.814	0.954	1239.032	1235.129	0.051	1214.761	1210.859	0.629	1282.202	1278.299	0.000
Beans	519.823	515.920	0.532	1019.932	1016.029	0.004	1048.091	1044.188	0.001	1005.110	1001.208	0.092
Cassava	585.320	581.417	0.132	1293.669	1289.766	0.000	1337.395	1333.492	0.000	1250.241	1246.338	0.000
Coffee	307.680	303.777	0.931	1054.181	1050.278	0.112	1091.141	1087.238	0.001	1036.031	1032.129	0.645
Sorghum	78.290	74.387	0.930	1053.391	1049.489	0.001	1076.397	1072.495	0.000	1045.201	1041.298	0.000
Sweet potato	134.892	130.989	0.901	1243.862	1239.959	0.000	1184.906	1181.003	0.000	1290.785	1286.882	0.000
Kenya—Beans	493.325	489.422	0.504	980.215	976.313	0.223	999.376	995.474	0.010	979.803	975.900	0.432
Coffee	308.382	304.479	0.721	1043.274	1039.371	0.109	1390.577	1386.674	0.000	1038.009	1034.106	0.323
Maize	352.530	348.627	0.756	1084.795	1080.893	0.332	1093.394	1089.491	0.112	1085.083	1081.180	0.532
Rice	785.164	781.262	0.303	1251.779	1247.876	0.121	1282.416	1278.513	0.012	1242.221	1238.318	0.231
Sugar Cane	771.239	767.336	0.944	1613.395	1609.492	0.007	1639.809	1635.906	0.000	1595.751	1591.848	0.321
Wheat	418.665	414.762	0.835	1153.186	1149.283	0.821	1160.712	1156.810	0.712	1162.411	1158.508	0.402
Somalia—Banana	306.400	302.498	0.889	1412.801	1408.899	0.000	1430.688	1426.786	0.000	1421.742	1417.839	0.000
Maize	577.876	573.973	0.811	1097.639	1093.737	0.943	1109.941	1106.038	0.423	1102.289	1098.386	0.434
Sorghum	299.456	295.554	0.954	978.390	974.487	0.131	976.162	972.259	0.203	1006.621	1002.718	0.015
Sugar Cane	1561.743	1557.841	0.252	1621.627	1617.724	0.009	1608.998	1605.095	0.019	1636.254	1632.351	0.003
Tanzania—Maize	602.620	598.718	0.632	1146.010	1142.107	0.100	1157.454	1153.551	0.005	1155.097	1151.194	0.103
Millet	357.070	353.168	0.922	1061.506	1057.604	0.966	1066.259	1062.356	0.533	1080.704	1076.801	0.121
Rice	645.884	641.981	0.532	1157.416	1153.513	0.802	1169.649	1165.746	0.242	1157.795	1153.893	0.499
Cotton Seed	480.611	476.708	0.332	999.445	995.543	0.854	1016.260	1012.358	0.166	999.811	995.908	0.664
Sorghum	264.040	260.138	0.834	1088.711	1084.809	0.211	1100.409	1096.506	0.023	1089.489	1085.586	0.512
Sweet potato	823.410	819.507	0.200	1303.455	1299.553	0.661	1307.969	1304.066	0.183	1305.698	1301.795	0.402
Uganda—Banana	269.292	265.389	0.909	1187.916	1184.013	0.005	1224.341	1220.438	0.000	1158.594	1154.691	0.443
Cassava	688.846	684.944	0.518	1361.801	1357.898	0.535	1361.721	1357.818	0.515	1369.707	1365.804	0.323
Coffee	614.601	610.698	0.833	1007.740	1003.837	0.907	1013.23	1009.328	0.516	1017.461	1013.558	0.420
Millet	700.174	696.271	0.003	1094.174	1090.271	0.074	1113.196	1109.294	0.008	1079.048	1075.145	0.187
Plantain	884.580	880.678	0.667	1245.936	1242.033	0.138	1242.476	1238.573	0.384	1261.018	1257.115	0.004
Sweet potato	618.508	614.605	0.654	1189.226	1185.324	0.087	1209.564	1205.662	0.012	1196.161	1192.258	0.065

**Table 4:** Continuation of Table 3.

Country—Crop	Power law			Lognormal			Fréchet			Stretched exponential		
	BIC	AICc	*p*–value	BIC	AICc	*p*–value	BIC	AICc	*p*–value	BIC	AICc	*p*–value
Rwanda—Beans	171.782	167.879	0.935	978.580	974.677	0.524	989.914	986.011	0.165	978.688	974.785	0.642
Cassava	152.243	148.340	0.909	1410.743	1406.840	0.019	1433.857	1429.954	0.004	1392.647	1388.744	0.176
Coffee	578.925	575.022	0.903	1017.965	1014.062	0.332	1040.145	1036.243	0.054	1022.827	1018.925	0.105
Potato	945.602	941.699	0.810	1332.154	1328.251	0.051	1363.027	1359.124	0.001	1324.835	1320.932	0.142
Sorghum	617.552	613.650	0.732	1038.192	1034.289	0.543	1054.371	1050.468	0.223	1043.405	1039.502	0.091
Sweet potato	278.219	274.317	0.983	1274.881	1270.979	0.675	1292.205	1288.303	0.263	1274.463	1270.560	0.243

**Fig 11:**
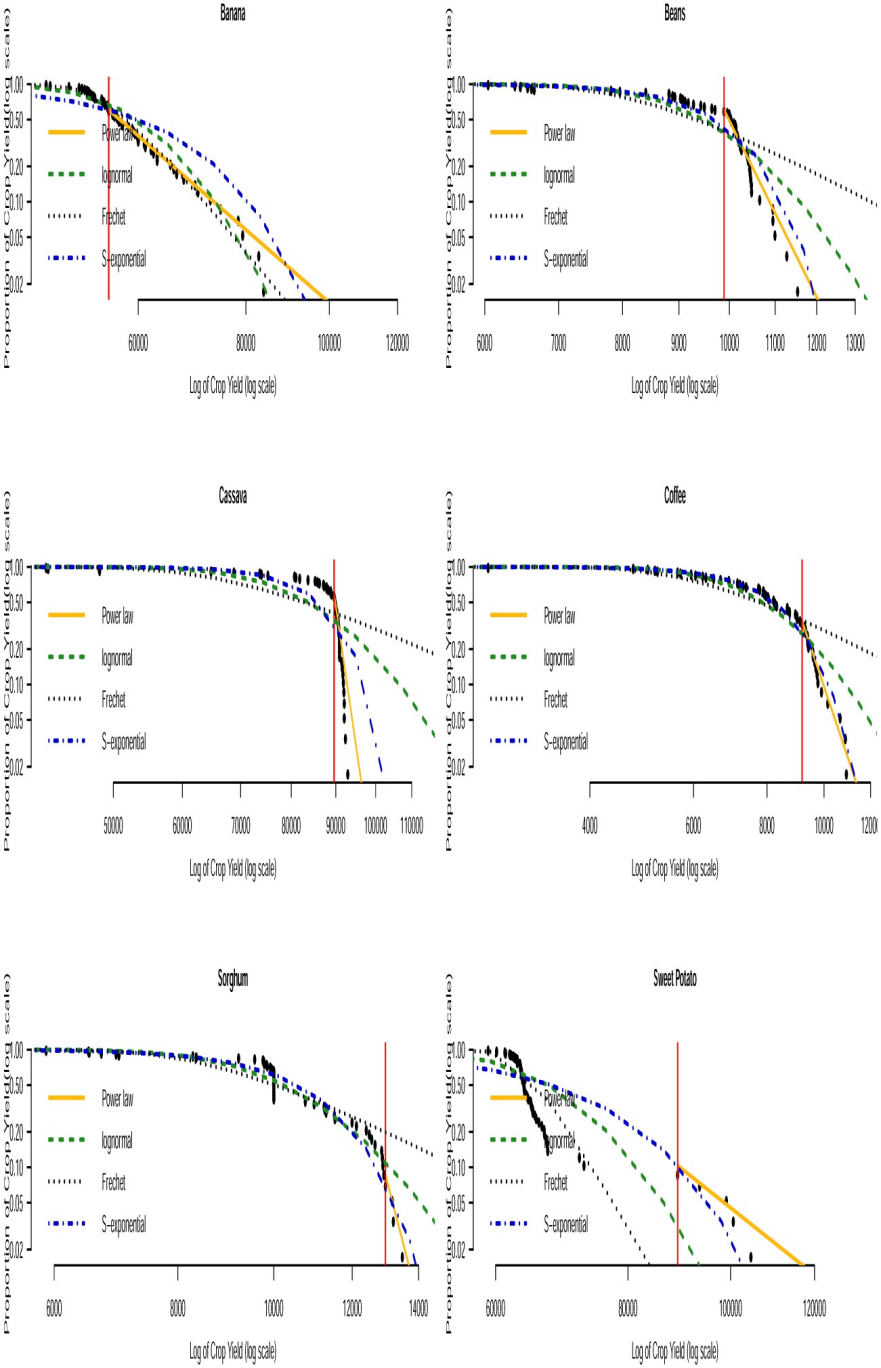
Log-log plots for crops yield in Burundi where the red line corresponds to the value of *x*_min_ in Table 7.

**Fig 12:**
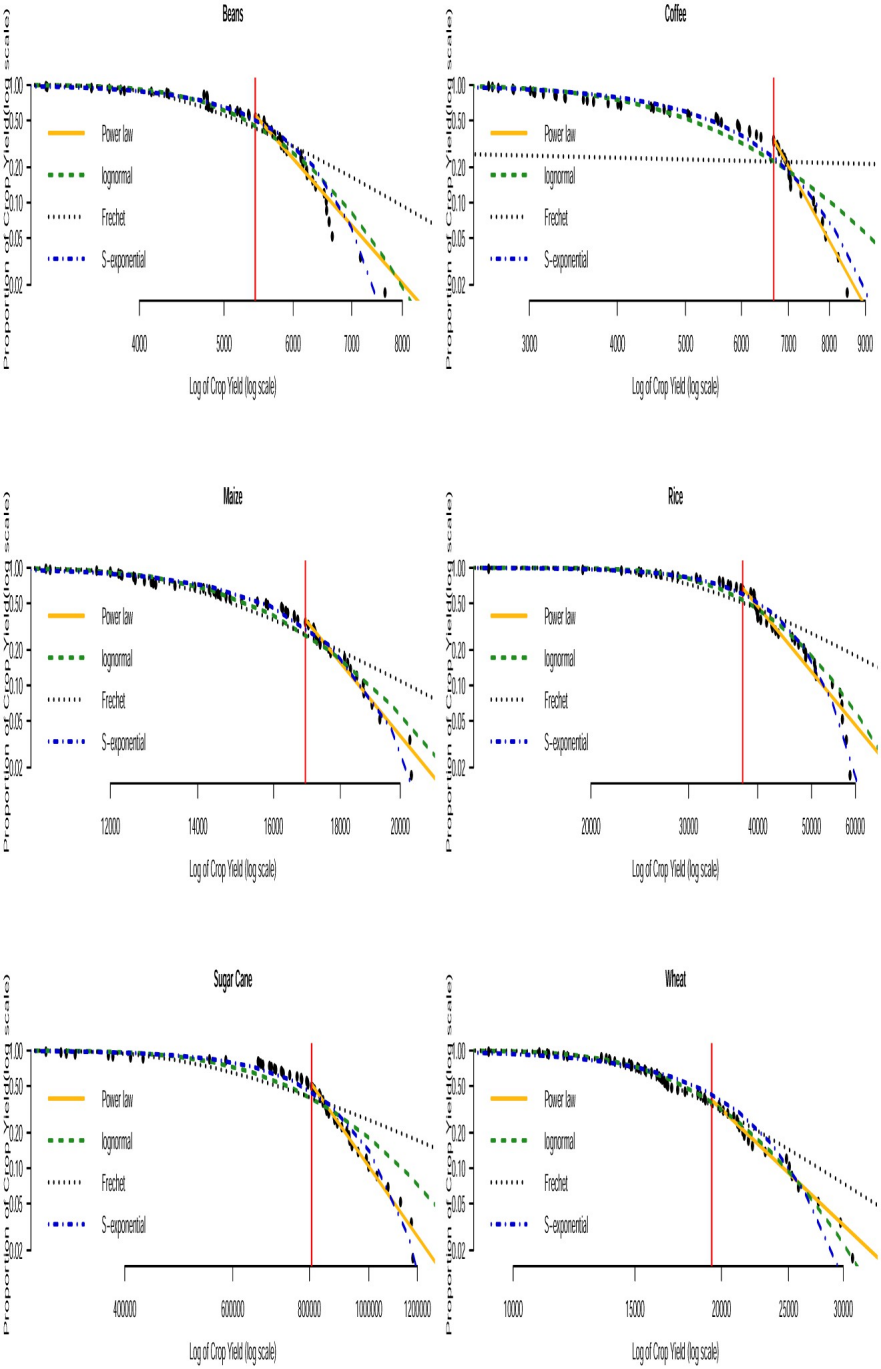
Log-log plots for crops yield in Kenya where the red line corresponds to the value of *x*_min_ in Table 7.

**Fig 13:**
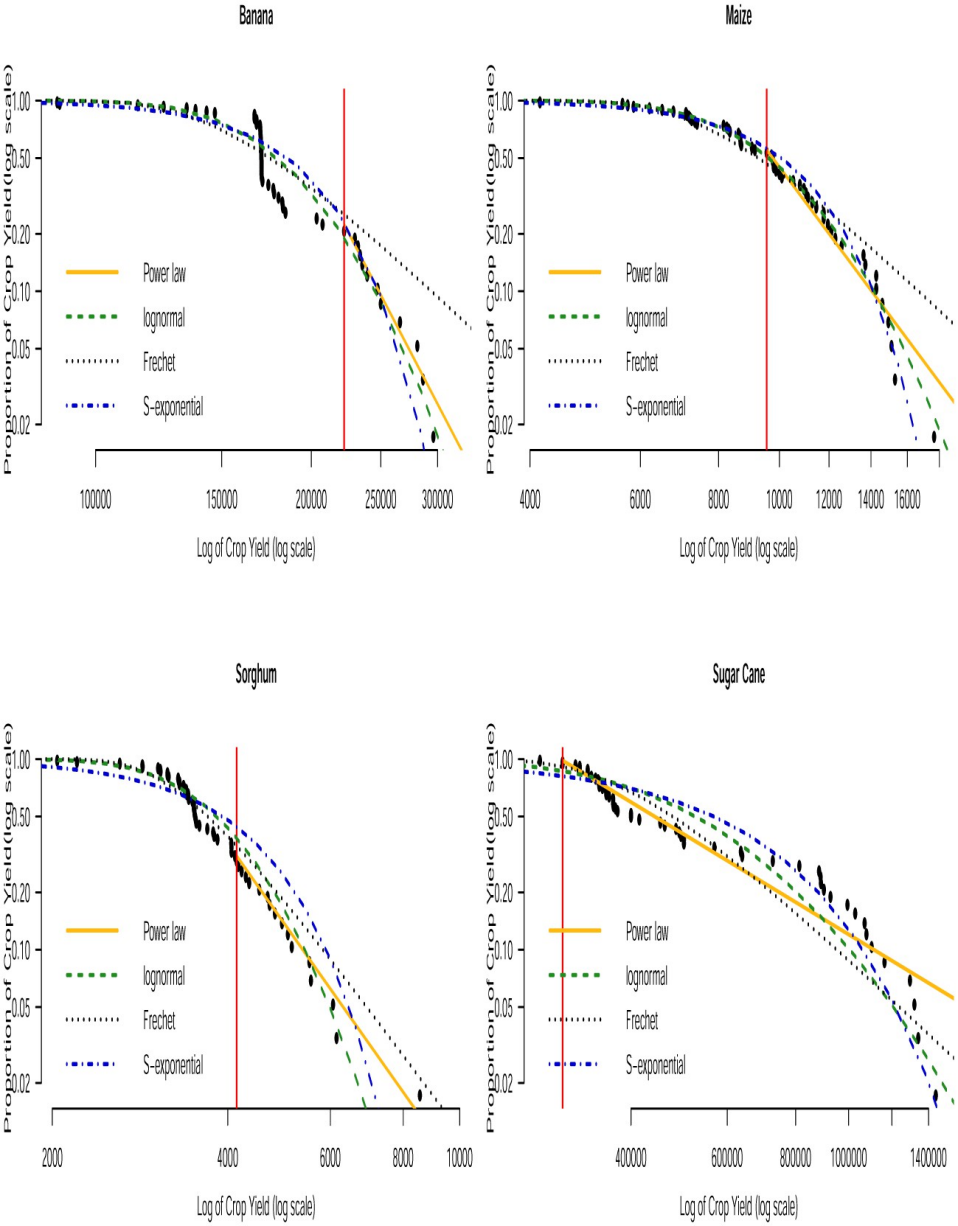
Log-log plots for crops yield in Somalia where the red line corresponds to the value of *x*_min_ in Table 7.

**Fig 14:**
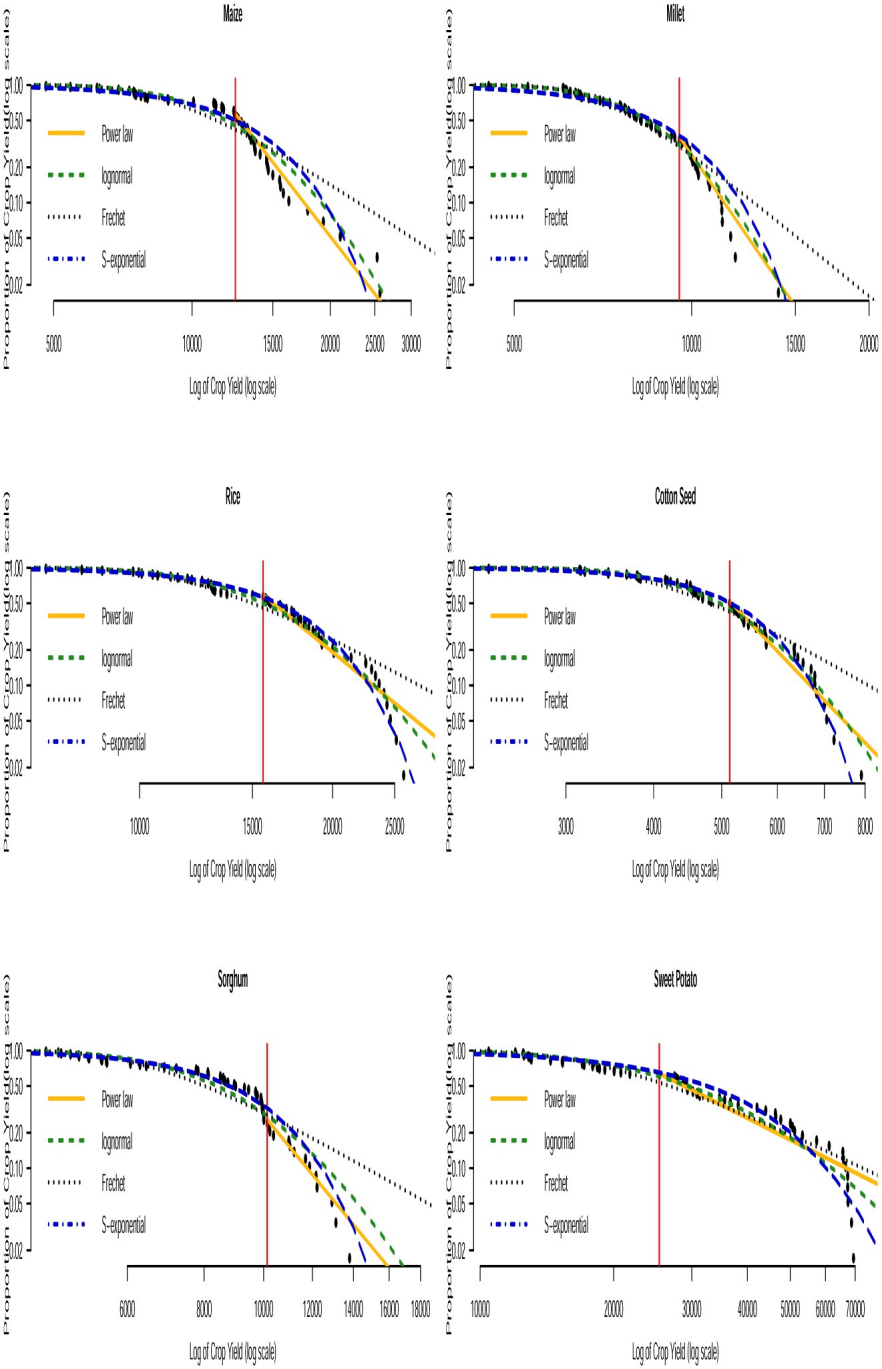
Log-log plots for crops yield in Tanzania where the red line corresponds to the value of *x*_min_ in Table 7.

**Fig 15:**
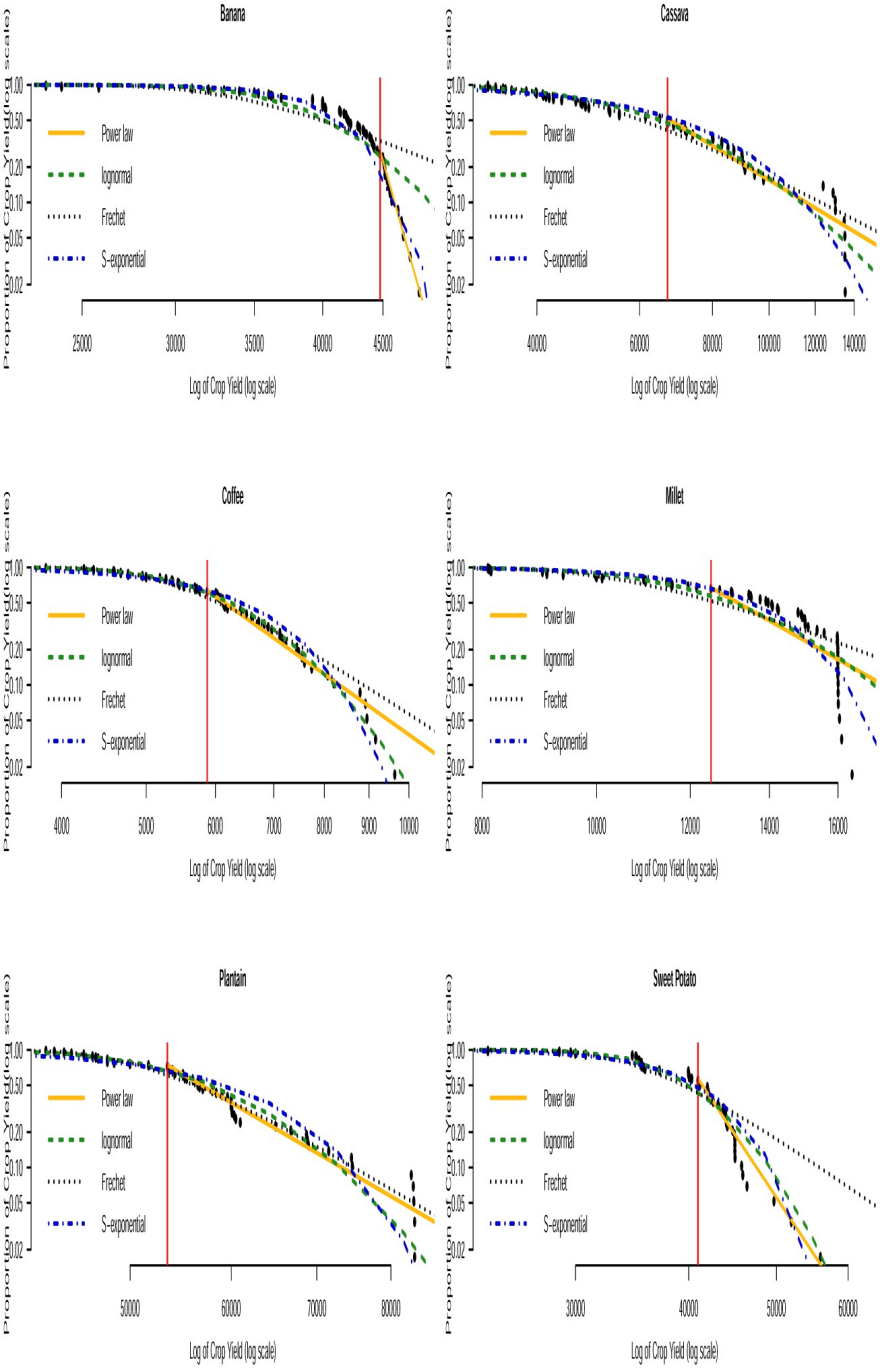
Log-log plots for crops yield in Uganda where the red line corresponds to the value of *x*_min_ in Table 7.

**Fig 16:**
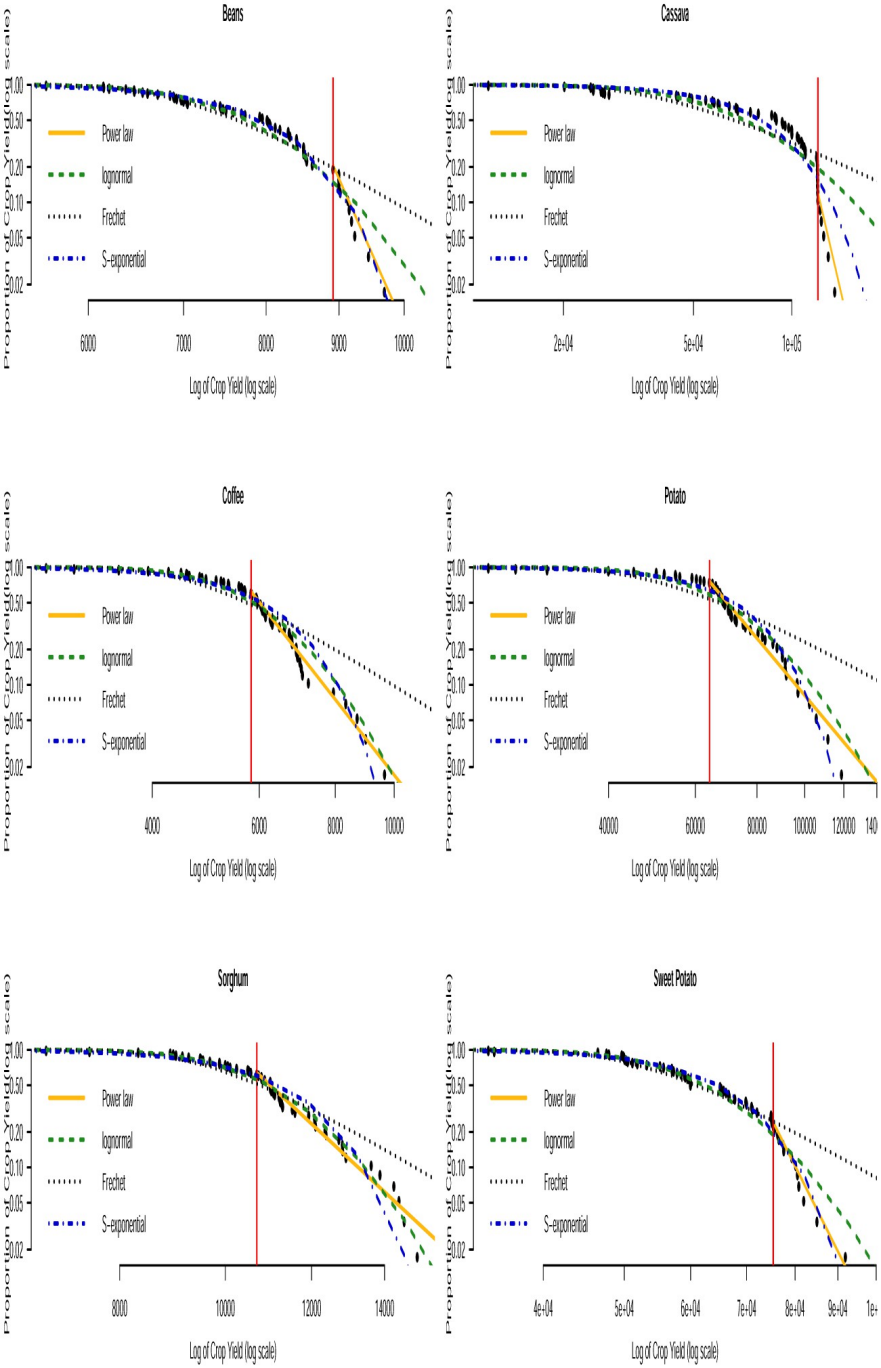
Log-log plots for crops yield in Rwanda where the red line corresponds to the value of *x*_min_ in Table 7.

Based on the AICc and BIC values in Tables 3 and 4, we can see that none of the three distributions that model the entire data (i.e. the lognormal, Fréchet and the stretched exponential distributions) consistently provide the best fit. None of them consistently gave the smallest AICc or smallest BIC values across the countries. The bootstrapped KS *p*–values in Table 5 indicate that the power law distribution is a plausible model for all the crop yield data. In general, the distribution with the smallest AICc and smallest BIC values corresponds to the distribution with the largest bootstrapped KS *p*–values. Fitting of such distributions to the tail of the data can be compared with that of the power law distribution by using Vuong’s test. The results of this comparison are presented in Table 6. We can observe that the stretched exponential distribution emerges as the best model for millet in Uganda and the power law distribution emerges as the best model for the rest of the crops except for a few cases where the winner is undecided. For instance, for sorghum in Burundi (power law and lognormal), sweet potato in Burundi (power law and Fréchet) and for banana in Somalia (power law and lognormal). The log-log plots of the fitted distributions superimposed with the empirical distributions are displayed in Figs 11 to 16. We can see that the power law distribution fits all the crop yield data well across the countries.

**Table 5:** Bootstrap KS-test *p*–values (as defined in Section 3) for the fitted distributions.

Country	Crop	Power law	Lognormal	Fréchet	Stretched exponential
Burundi	Banana	0.966	0.732	0.925	0.094
	Beans	0.902	0.424	0.112	0.732
	Cassava	0.832	0	0	0.021
	Coffee	0.943	0.831	0.322	0.941
	Sorghum	0.952	0.305	0.101	0.324
	Sweet potato	0.903	0	0.221	0
Kenya	Beans	0.913	0.902	0.591	0.944
	Coffee	0.928	0.821	0	0.922
	Maize	0.929	0.931	0.820	0.954
	Rice	0.805	0.821	0.461	0.844
	Sugar Cane	0.945	0.611	0.132	0.940
	Wheat	0.905	0.962	0.901	0.922
Somalia	Banana	0.923	0.016	0.012	0
	Maize	0.933	0.942	0.912	0
	Sorghum	0.955	0.813	0.828	0
	Sugar Cane	0.903	0.523	0.631	0
Tanzania	Maize	0.956	0.787	0.511	0.723
	Millet	0.931	0.962	0.936	0.902
	Rice	0.902	0.951	0.921	0.952
	Cotton Seed	0.933	0.971	0.822	0.949
	Sorghum	0.939	0.827	0.609	0.906
	Sweet potato	0.804	0.965	0.919	0.915
Uganda	Banana	0.965	0.514	0.055	0.931
	Cassava	0.901	0.961	0.919	0.924
	Coffee	0.955	0.980	0.911	0.923
	Millet	0.201	0.612	0.423	0.919
	Plantain	0.932	0.831	0.925	0.506
	Sweet potato	0.909	0.810	0.424	0.734
Rwanda	Beans	0.958	0.951	0.901	0.911
	Cassava	0.987	0.612	0.301	0.812
	Coffee	0.943	0.902	0.613	0.832
	Potato	0.924	0.732	0.321	0.815
	Sorghum	0.932	0.933	0.841	0.822
	Sweet potato	0.949	0.910	0.906	0.931

**Table 6:** Vuong test statistic (Λ) and its *p*–value for comparing the upper tail (i.e. *x* > *x*_min_) of the fitted power law distribution and the best among the rest of the competing distributions.

Country	Crop	Contest	Statistic (Λ)	*p*–value	Winner
Burundi	Banana	Power law *vs* Fréchet	2.6671	0.0077	Power law
	Beans	Power law *vs* Stretched exponential	6.7366	0	Power law
	Cassava	Power law *vs* Stretched exponential	10.4399	0	Power law
	Coffee	Power law *vs* Stretched exponential	4.1021	0	Power law
	Sorghum	Power law *vs* Lognormal	1.2454	0.2130	Undecided
	Sweet potato	Power law *vs* Fréchet	0.7442	0.4568	Undecided
Kenya	Beans	Power law *vs* Stretched exponential	3.9727	1.0×10^−4^	Power law
	Coffee	Power law *vs* Stretched exponential	3.5192	4.0×10^−4^	Power law
	Maize	Power law *vs* Stretched exponential	2.8513	0.0044	Power law
	Rice	Power law *vs* Stretched exponential	4.4303	0	Power law
	Sugar Cane	Power law *vs* Stretched exponential	4.3048	0	Power law
	Wheat	Power law *vs* Lognormal	2.5905	0.0096	Power law
Somalia	Banana	Power law *vs* Lognormal	1.7339	0.0829	Undecided
	Maize	Power law *vs* Lognormal	2.6642	0.0077	Power law
	Sorghum	Power law *vs* Fréchet	1.9648	0.0494	Power law
	Sugar Cane	Power law *vs* Fréchet	4.5875	0	Power law
Tanzania	Maize	Power law *vs* Lognormal	5.4006	0	Power law
	Millet	Power law *vs* Lognormal	3.8995	1.0×10^−4^	Power law
	Rice	Power law *vs* Stretched exponential	2.8193	0.0048	Power law
	Cotton Seed	Power law *vs* Lognormal	2.8774	0.0040	Power law
	Sorghum	Power law *vs* Stretched exponential	2.8893	0.0039	Power law
	Sweet potato	Power law *vs* Lognormal	2.3193	0.0204	Power law
Uganda	Banana	Power law *vs* stretched exponential	2.9194	0.0035	Power law
	Cassava	Power law *vs* Lognormal	2.4766	0.0133	Power law
	Coffee	Power law *vs* Lognormal	2.7274	0.0064	Power law
	Millet	Power law *vs* Stretched exponential	−2.8155	0.0049	Stretched exponential
	Plantain	Power law *vs* Fréchet	3.1373	0.0017	Power law
	Sweet potato	Power law *vs* Lognormal	3.9022	1.0×10^−4^	Power law
Rwanda	Beans	Power law *vs* Stretched exponential	3.5970	3.0×10^−4^	Power law
	Cassava	Power law *vs* Stretched exponential	2.9573	0.0031	Power law
	Coffee	Power law *vs* Lognormal	4.3420	0	Power law
	Potato	Power law *vs* Stretched exponential	5.4570	0	Power law
	Sorghum	Power law *vs* Lognormal	4.1122	0	Power law
	Sweet potato	Power law *vs* Lognormal	2.6648	0.0077	Power law

Since the power law model appears to be a plausible distribution for virtually all the crops across countries, we present the estimate for the parameters of the distribution in Table 7. We see that the power law mechanism may occur at varying degrees depending on the type of crop and country. See the *n*_tail_ values for crops in Table 7, where *n*_tail_ denotes the total number of observations equal to or above the threshold value *x*_min_, i.e. the total number of data points following the power law distribution. The occurrence of such extremely high crop yield definitely has positive impact on farmers and food security. In this case, farmers can make huge profits. Crop yield risk insurance policies for such crops can attract relatively lower premium rates compared to crops with lower yields. The *α* value of the fitted power law model describes the heaviness of the tail distribution corresponding to extremely high crop yield events with yield > *x*_min_. According to Table 7, the estimates of *α* are all > 2 indicating that the data in the right tail of the distribution show significant high inequality (i.e. large crop yield). However, there are two special cases satisfying 2 < *α* ≤ 3 specifically in Somalia for sugar cane and Tanzania for sweet potato. In these cases, the variance and higher-order moments for the crop yields are infinite regardless of whether their mean yield exists or not. Hence, the classical central limit theorem does not hold for these yield data. The consequence of the infinite variance and higher order moments is that empirical estimates of the means converge very slowly due to the regular occurrence of extremely large crop yield values. These characteristics suggest that crop harvest with extremely large yield could sometimes occur for sugar cane in Somalia and sweet potato in Tanzania. Such events could often be of great importance to the farmers and other investors in agribusiness. This behavior is referred to as the black swan mechanism (see [67]). The black swan mechanism describes events coming as a surprise. It has a major effect (positive or negative) and is often inappropriately rationalized. Farmers can have the tendency to break even and even enjoy lower crop yield risk insurance policies in Somalia and Tanzania if they invest in sugar cane and sweet potato, respectively, due to their potential for extremely high yield.

**Table 7:** Parameter estimates for the power law distribution for all the crop yield data sets (*x*_min_ and *α* are parameters of the power distribution; *α*_se_ is the standard error corresponding to *α*; *n*_tail_ is the number of data exceeding *x*_min_).

Country	Crop	*n*	*n*_tail_	*x*_min_	*α*	*α*_se_
Burundi	Banana	58	35	55455	7.4026	1.0822
	Beans	58	35	9888	19.9503	3.2032
	Cassava	58	35	89591	52.1287	8.7685
	Coffee	58	35	9180	15.9219	3.3366
	Sorghum	58	5	12964	33.5816	14.5710
	Sweet potato	58	6	89127	8.1898	2.9352
Kenya	Beans	58	32	5429	9.4680	1.4969
	Coffee	58	20	6669	11.9168	2.4411
	Maize	58	21	16922	14.5952	2.9667
	Rice	58	39	37568	6.7330	0.9180
	Sugar Cane	58	30	806694	8.5033	1.3699
	Wheat	58	22	19359	6.5818	1.1901
Somalia	Banana	58	13	222222	8.2251	2.0039
	Maize	58	32	9544	5.4249	0.7822
	Sorghum	58	18	4143	5.3367	1.0222
	Sugar Cane	58	57	300000	2.7445	0.2311
Tanzania	Maize	58	33	12427	6.0445	0.8781
	Millet	58	21	9530	8.3193	1.5972
	Rice	58	34	15583	5.4751	0.7675
	Cotton Seed	58	30	5132	7.2276	1.1370
	Sorghum	58	15	10133	7.3606	1.6423
	Sweet potato	58	37	25323	2.9081	0.3137
Uganda	Banana	58	16	44748	36.7026	8.9257
	Cassava	58	30	66988	4.0035	0.5484
	Coffee	58	37	5873	6.3153	0.8738
	Millet	58	39	12496	6.6790	0.9094
	Plantain	58	43	53474	7.3752	0.9722
	Sweet potato	58	33	40960	12.6778	2.0328
Rwanda	Beans	58	12	8914	28.4564	7.9260
	Cassava	58	7	120265	13.0159	4.5416
	Coffee	58	36	5817	7.6296	1.1049
	Potato	58	44	64088	6.0225	0.7572
	Sorghum	58	37	10688	9.6451	1.4212
	Sweet potato	58	14	75333	15.2064	3.7968

All the estimated *α* values for the power law distribution in Table 7 are > 3 except for sugar cane in Somalia and sweet potato in Tanzania. This indicates that the sample means for these crops are Gaussian distributed and that their variances are finite. Hence, the standard central limit theorem applies for these crop yield data. The finite mean and variance and the observed evidence of underdispersion in Table 1 suggest that east African regional food security does not seem to be extremely volatile as regular crop yields for these crops tend to cluster around the mean crop yield.

Ignoring the impacts of climate and environment, soil structures and compositions/nutrients, crop species, mechanization and technology, etc on crop yields, the observed black swan behaviour for the yields of sugar cane in Somalia and sweet potato in Tanzania could be explained by the so called “*rich getting richer*” principle or the “*preferential attachment*” principle. Based on these principles, these two crops have potentials for extremely high yield perhaps because of either high demand (so every farmer tends to make them their choice crops for cultivation) or common practice such as irrigation adopted by all the farmers being capable of increasing crop yield [36]. So, speaking of crop harvest, yield could follow the pattern of the rich getting richer or the preferential attachment principle. The extremely high yields for sugar cane in Somalia and sweet potato in Tanzania are not just a little bit higher than the normal yield for the same or different crops in the same or other countries. Instead they are so much higher that they cause their distributions to skew significantly.

## 5 Conclusions

We have analyzed the trend and tail of some yearly crop yield data such as banana, plantain, beans, cassava, coffee, sorghum, potato, sweet potato, maize, rice, sugar cane, wheat, millet and cotton seed from 1961 to 2018 in six east African countries: Burundi, Kenya, Somalia, Tanzania, Uganda and Rwanda. An exploratory analysis of the crop yield data reveals three structural patterns in each of the series. They are: increasing, decreasing and stagnant trends. Ten years (2019–2028) time series point forecast based on the fitted ARIMA models shows that majority of the crops will experience stagnant yield in different countries with only sorghum and coffee showing the tendency for significant and persistent upward trend in Burundi and Rwanda, respectively, while beans indicates significant and persistent yield decrease in Burundi, Kenya and Rwanda.

We used the power law, lognormal, Fréchet and stretched exponential distributions to describe high yields in all the crops across the countries. Based on Vuong’s test, we observed that the stretched exponential distribution gave the best fit for millet in Uganda while the power law distribution gave the best fit for the other crops except for a few undecided cases. The log-log plots were used to visually inspect the performance of the fitted distributions. The power law distribution appeared to fit the upper tail of all the crop yield data better than the other distributions in all the countries. Based on the estimated *α* value of the fitted power law model, we found potential for extremely high yield in sugar cane in Somalia and sweet potato in Tanzania indicating the inappropriateness of the Gaussian distribution for describing these crop yields. Other crops in Burundi, Kenya, Somalia, Tanzania, Uganda and Rwanda can produce only high but not extremely high yields. Though the time series point forecasts for majority of the crops show yield stagnancy with a few exceptions, the evidence from the power law analysis indicates the potential for high yield for all the crops and provides specific calibrations for the yield of all the crops in terms of what quantity of yield is considered high.

We characterize the evidence for extremely high yield for sugar cane and sweet potato in Somalia and Tanzania, respectively, as black swan where the “*rich getting richer*” or the “*preferential attachment*” could be the underlying generating process, meaning that either the two crops are increasingly at lower risk of climate change and environmental challenges such as being drought resistant or farmers are constantly doing many things right (such as adopting favorable planting strategies, large crop areas, etc) as far as the cultivation of the two crops are concerned in the two countries.

ARIMA(0,1,1) was used to model and predict coffee in Burundi and Kenya; beans in Burundi; wheat in Kenya; rice, cotton seed, and sweet potato in Tanzania; millet in Uganda. ARIMA(2,1,0) was used to model and predict sorghum in Tanzania. ARIMA(2,1,2) was used to model and predict banana in Burundi. ARIMA(0,1,2) was used to model and predict banana in Uganda and potato in Rwanda. ARIMA(0,1,0) was used to model and predict cassava in Burundi and Uganda; sugarcane in Kenya and Somalia; rice in Kenya; maize in Somalia; plantain and sweet potato in Uganda. ARIMA(1,0,0) was used to model and predict Sorghum in Burundi and Rwanda; beans in Kenya and Rwanda; millet in Tanzania; coffee in Uganda. ARIMA(2,1,1) was used to model and predict sweet potato in Burundi. ARIMA(0,1,3) was used to model and predict maize in Kenya. ARIMA(0,0,4) was used to model and predict banana in Somalia. ARIMA(1,1,1) was used to model and predict sorghum in Somalia; maize in Tanzania. ARIMA(2,0,2) was used to model and predict sweet potato in Rwanda.

The yield forecast in Burundi shows an initial quick decline in 2019 followed by an increase for banana; a sharp increase in 2019 followed by an increase for sweet potato; sorghum shows a quick increase from 2019 to 2028; beans shows a sharp decrease from 2019 to 2028; neither cassava nor coffee show any tendency to increase or decrease from 2019 to 2028. The forecast of the crop yield in Kenya indicates continuous decline of beans yield from 2019 to 2028; no decrease or increase pattern in yield is evident for coffee, rice, wheat and sugar cane from 2019 to 2028; maize shows a sharp decline in 2019 with an immediate increase followed by a stable trend. In Somalia, the yield forecast for maize and sugar cane does not indicate any pattern; banana shows an initial moderate increase in 2019 followed by the lack of pattern until 2028; sorghum experienced a sharp drop in 2019 followed by a period of no trend up to 2028. The yield forecast in Tanzania indicates no significant trend for maize, rice, sweet potato and cotton seed for the whole forecast period; millet is slightly decreased in 2019 and remained stagnant until 2028. The yield forecast in Uganda indicates that banana, cassava, millet, plantain and sweet potato did not show any significant pattern from 2019 to 2028; coffee shows a slight increase in 2019 followed by a period of no change in yield. The yield forecast in Rwanda indicates that beans persistently decreased from 2019 to 2028; sweet potato shows initial increase followed by a slow decrease; coffee indicated an upward trend from 2019 to 2028; cassava, potato and sorghum did not show any significant pattern.

In our discussion in Section 4, we saw how the literature points in the direction of climate change as the major cause of the observed yield stagnancy and decline. On this backdrop, we suggest that a promising future in favour of high crop yield could await east Africa if urgent changes or improvements on the cropping systems and infrastructures that currently exist in east Africa could be made in order to meet up with the inevitable future demand of agricultural produce due to the increasing population and the challenge of negative impacts of climate change. Science and technology could be useful in showing how agricultural production can be significantly improved in east Africa. For instance, the construction of irrigation systems and rainwater harvesting structures could help cushion the impact of climate change.

Further, various climate adaptation/smart strategies could be adapted to increase yields in east Africa. According to [68], short-duration pigeon pea varieties developed by the International Crops Research Institute for Semi-Arid Tropics and the Kenya Agricultural Research Institute can give high yields and escape drought, but require non-traditional management practices (for example, sole-cropping, spraying against insect pests). According to [69], NERICA, a new rice for Africa, has shown high potential to revolutionize rice farming, producing high yield with minimum inputs in stress-afflicted ecologies. [70] observed that cassava mosaic disease (CMD) resistant cassava varieties released in western Kenya and Uganda yielded up to three times more than local varieties. [71] demonstrated that high yields of maize were recorded from certain varieties (Pwani Hybrid 4-PH4, Coast Composite Maize-CCM and the local check-Mdzihana) but they usually required relatively high rainfall amounts in order for them to produce better yields. [72] showed that increased knowledge of varieties, environment and management factors can double total yield of maize, sorghum, millet and groundnut from 1.67 to 3.29 tons per hectare from the average 5.1 hectares that farmers usually crop in south east Zimbabwe. [73] showed that improved maize varieties outyielded the traditional control variety by 26–46% across sites and season in central Mozambique. [74] showed that the use of organic soil management practices such as reduced tillage, mulching and leguminous crops in the northern part of Tanzania increased the production of food crops from an average of 0.5 ton per hectare to 1.5 ton per hectare; subsequently, maize yields increased from 12,000 kilogram to 20,000 kilogram per 4.8 hectares. [75] suggested that relaxing liquidity constraints could help to encourage farmers’ adaptation through the implementation of soil, water and land management strategies; thereby, positioning east Africa for food sufficiency in the face of the current global food crisis. [76] noted that intensive manuring with a combination of green and poultry manure produced high yields of maize in central Uganda that were comparable to those with mineral fertilizers. [77] demonstrated that households in Kenya adapting to climate change and climate variability through uptake of technologies such as early planting, use of improved crop varieties, and crop diversification produced 4877 kilograms of maize yield equivalent / hectare per year against 3238 kilograms of maize yield equivalent / hectare per year for households that did not adapt (a 33.6% difference between the two groups). [78] found that fertilizer application in the intercropping system is eastern and southern Africa improved cereal yields by 71–282% and pigeon pea yields by 32–449%, increased benefit-cost ratios by 10–40%, and reduced variability in cereal yields by 40–56% and pigeon pea yields by 5–52% compared with unfertilized intercrops. [79] showed that drought resistant climate-smart maize hybrids in Kenya increased yields 33 to 54% relative to conventional hybrids. According to [80], climate adaptation strategies in the central highlands of Kenya included the use of fertilizer and manure in combination (71%), terracing (66%), and crop rotation (60%). [81] showed that climate-smart adaptation practices significantly enhanced wheat yield by 34.35% in southern Ethiopia. [82] showed that use of mulching and permanent planting basin dimensions on maize in western Uganda relatively increased yield by 11–66% and water use efficiency by 33–94% compared to conventional practices.

The findings in this paper underscore the importance of using climate-smart agricultural alternatives to improve resilience farming system and the livelihood of subsistence farmers due to the impact of climate change in east Africa. Currently, crop yield for majority of the crops in different countries has been confirmed to neither increase nor decrease with only few crops experiencing all time increase or decrease in yield. Urgent attention should be paid to beans production in the affected countries in order to reverse the persistent downward trend of its yield. This paper brings good news of hope for crop yield increase in east Africa if adaptive farming methods and strategies are adequately harnessed in the region in the face of climate and environmental challenges and rising global demand for agricultural produce.

The data from 1961 to 2018 consist of only 58 observations. Hence, the results and forecasts in this paper should be treated conservatively. A future work is to see if more frequent and more up-to-date data are available. Another is to consider multivariate modelling of yield by considering country and crop. The disadvantage of the length of the observed series can be interpolated by explaining the common factor for each country and crop.

## Supporting information

S1 Data (CSV)

S2 Data (CSV)

S3 Data (CSV)

S4 Data (CSV)

S5 Data (CSV)

S6 Data (CSV)

S7 Data (CSV)

S8 Data (CSV)

S1 File (TXT)
